# adverSCarial: assessing the vulnerability of single-cell RNA-sequencing classifiers to adversarial attacks

**DOI:** 10.1093/bioinformatics/btaf168

**Published:** 2025-04-15

**Authors:** Ghislain Fievet, Julien Broséus, David Meyre, Sébastien Hergalant

**Affiliations:** INSERM U1256, Nutrition, Genetics, and Environmental Risk Exposure (NGERE), University of Lorraine, Nancy, 54500, France; INSERM U1256, Nutrition, Genetics, and Environmental Risk Exposure (NGERE), University of Lorraine, Nancy, 54500, France; Department of Biological Hematology, Laboratory Center, University Hospital of Nancy, Nancy, 54500, France; INSERM U1256, Nutrition, Genetics, and Environmental Risk Exposure (NGERE), University of Lorraine, Nancy, 54500, France; Department of Molecular Medicine, Division of Biochemistry, Molecular Biology, and Nutrition, University Hospital of Nancy, Nancy, 54500, France; INSERM U1256, Nutrition, Genetics, and Environmental Risk Exposure (NGERE), University of Lorraine, Nancy, 54500, France

## Abstract

**Motivation:**

Several machine learning (ML) algorithms dedicated to the detection of healthy and diseased cell types from single-cell RNA sequencing (scRNA-seq) data have been proposed for biomedical purposes. This raises concerns about their vulnerability to adversarial attacks, exploiting threats causing malicious alterations of the classifiers’ output with defective and well-crafted input.

**Results:**

With adverSCarial, adversarial attacks of single-cell transcriptomic data can easily be simulated in a range of ways, from expanded but undetectable modifications to aggressive and targeted ones, enabling vulnerability assessment of scRNA-seq classifiers to variations of gene expression, whether technical, biological, or intentional. We exemplify the usefulness and performance with a panel of attack modes proposed in adverSCarial by assessing the robustness of five scRNA-seq classifiers, each belonging to a distinct class of ML algorithm, and explore the potential unlocked by exposing their inner workings and sensitivities on four different datasets. These analyses can guide the development of more reliable models, with improved interpretability, usable in biomedical research and future clinical applications.

**Availability and implementation:**

*adverSCarial* is a freely available R package accessible from *Bioconductor*: https://bioconductor.org/packages/adverSCarial/ or https://doi.org/10.18129/B9.bioc.adverSCarial. A development version is available at https://github.com/GhislainFievet/adverSCarial.

## 1 Introduction

Adversarial attacks represent a significant threat to machine learning (ML) tools (Goodfellow *et al.* 2015). These attacks have been investigated in various medical data fields, such as X-rays (Zhou *et al.* 2021), electrocardiograms (Han *et al.* 2020), and tissue slide analyses (Ghaffari Laleh *et al.* 2022). On single-cell-transcriptomic-data, their potential impact has been studied on white-box gradient-based ML algorithms (Sadria *et al.* 2023) but remains under-evaluated for other methods. As scRNA-seq cell type classifiers hold high potential for routine clinical applications, understanding their vulnerabilities to such attacks is increasingly popular (González-Silva *et al.* 2020, Su *et al.* 2022). Fast Gradient Sign Method (FGSM) and Projected Gradient Descent (PGD) are the two adversarial white-box methods that have been applied on scRNA-seq data (Sadria *et al.* 2023). FGSM is a single-step loss-minimizing function which uses the sign of the existing gradient with respect to the input data to create perturbations that push predictions away from the originally predicted class (Goodfellow *et al.* 2015). The size of the step, or attack level, is set by the parameter *ε*:

(1) $$x_{ad\upsilon }=x+\epsilon \;\mathrm{sign}\left(\nabla_{x}L\left(f\left(x\right),y\right)\right)$$

PGD iteratively applies small perturbations in the gradient of the loss’ direction, with respect to the input data. It is followed by a projection step to ensure the perturbation remains in the same range (Madry *et al.* 2019). Here the step size is set with each iteration:

(2) $$x_{N+1}^{ad\upsilon }=\mathrm{Pro}j_{x,\epsilon }\;\{x_{N}^{ad\upsilon }+\epsilon \;\mathrm{sign}\;\left(\nabla_{x}J\left(x_{N}^{ad\upsilon },y_{\mathrm{true}}\right)\right)\}$$

While these approaches target white-box gradient-based methods such as deep learning models, most scRNA-seq classifiers rely on different algorithms (Zhao *et al.* 2020), such as random forest (Tan and Cahan 2019), support vector machines (Nguyen and Griss 2022), XGBoost (Lieberman *et al.* 2018), and statistical models (Zhang *et al.* 2019): this singularity urges for the development of new, specialized strategies. Given the time-intensive nature of cell classification processes, these novel attack modes should be computationally cheap. Beyond the immediate security concerns, they should also be able to provide help in interpreting the generated models and offer insights into their decision-making layers.

*adverSCarial* features specific scRNA-seq adversarial attack algorithms: two of these attacks cause cell misclassifications with switching on/off unique genes or with imperceptibly modifying several genes, another one modifies large groups of genes without altering cell labeling, and another one implements a novel gradient descent algorithm adapted to cell clusters and black-box models. The full set of functions enables the investigation of single-cell transcriptomic classifiers under a dual security/transparency scrutiny, within a development frame that is both time- and resource-efficient. Here we exemplify the use, usefulness, and performance of *adverSCarial* by assessing the robustness of five scRNA-seq classifiers, each belonging to a distinct class of ML algorithm: *scType* (marker-based) (Ianevski *et al.* 2022), *CHETAH* (hierarchical clustering) (de Kanter *et al.* 2019), *scAnnotatR* [support vector machine (SVM)] (Nguyen and Griss 2022), *scRF* [random forests (RF)] (Breiman 2001), and *scMLP* [multilayer perceptron (MLP)] neural networks (Almeida 2020).

## 2 Materials and methods

### 2.1 Datasets

Four distinct scRNA-seq datasets were used in this study:

- PBMC3k is commonly used in benchmarks and is composed of 2700 peripheral blood mononuclear cells plus a few residual platelets and dendritic cells from a healthy human donor, already filtered for poor quality cells (scRNA expression dataset by Cell Ranger v1.1.0, Illumina NextSeq 500, 10x Genomics, https://cf10xgenomics.com/samples/cell/pbmc3k/pbmc3k_filtered_gene_bc_matrices.tar.gz). PBMC3k contains cells with distinct or with similar transcriptomic profiles, such as B cells and CD4 T cells, or classical (CD14+) and non-classical (FCGR3A+) monocytes, respectively.
- AXILLA10k includes 10 623 annotated cells from breast cancer metastatic core biopsies sampled within the axillary lymph node of a single patient (Klughammer *et al.* 2024) (https://cellxgene.cziscience.com/e/12c868c6-94df-48b5-acf2-b82f6aa14074.cxg/). AXILLA10k contains T cells, blood vessel endothelial cells, blood vessel smooth muscle cells, fibroblasts, macrophages, and malignant cells.
- LIVER10k includes 10 016 annotated cells from breast cancer metastatic core biopsies sampled within the liver of a single patient (Klughammer *et al.* 2024) (https://cellxgene.cziscience.com/e/cd6398a9-c0af-4467-9091-c536866535bd.cxg/). LIVER10k contains blood vessel smooth muscle cells, endothelial cells, macrophages, malignant cells, mature NK T cells, and monocytes. Underrepresented cell types (7 blood vessel endothelial cells and 9 fibroblasts) were removed.
- KIDNEY10k includes 10 790 annotated cells from fetal nephrons of 6 different donors (Stewart *et al.* 2019) (https://cellxgene.cziscience.com/e/08073b32-d389-41f4-a4fd-616de76915ab.cxg/). KIDNEY10k contains proximal tubule epithelial cells, kidney cells, kidney epithelial cells, loop of Henle cells, mesenchymal cells and podocytes.

Depending on the scRNA-seq model used, multiple datasets were prepared from the aforementioned sources following the procedure provided with *Seurat* (v5.0.3), which includes a standardized pipeline for further filtering, scaling, clustering and annotating the data (Hao *et al.* 2024). These quality control and preprocessing steps were distinct for each scRNA-seq dataset and strictly depended on the thresholds applied by the original authors in their inclusion/exclusion protocols. Specifically, cells with abnormally low or abnormally high numbers of genes were excluded (<200 and >2500 for PBMC3k, <500 for KIDNEY10k, <8000 for LIVER10k, and AXILLA10k), as well as cells with a high proportion of mitochondrial gene expression (>5% for PBMC3k, >20% for KIDNEY10k, >50% for LIVER10k and AXILLA10k). Raw data were log-normalized and scaled. Variable genes were selected with the *FindVariableGenes* function in *Seurat* for PBMC3k and KIDNEY10k (Stewart *et al.* 2019, Hao *et al.* 2024), or following the *Scanpy* (v1.7.2) workflow for AXILLA10k and LIVER10k (Klughammer *et al.* 2024), as detailed in the original publications. PCA and UMAP were then applied as dimensional reduction algorithms.

- *Undivided sets* contain all annotated cells.
- *Training sets* contain half of the cells, proportionally representing each of the cell types.
- *Test/validation sets* contain about half of the cells not overlapping with the training set cells and proportionally representing each of the cell types.
- *Balanced training sets* are alternatives for training sets used with RF and MLP algorithms and comprise 100 randomly selected cells per cell type. When the initial number of cells was <100, a bootstrap sampling on gene expression values within each relevant cell type generated additional ones.

PBMC3k was annotated by clustering the cells with the *FindClusters* function (a KNN graph-based approach), then for each cluster, each cell type was identified using a set of marker genes. For AXILLA10k, LIVER10k, and KIDNEY10k, cell annotations were provided directly with the datasets.

### 2.2 scRNA-seq classifier preparation

For *scType*, *CHETAH* and *scAnnotatR*, we prepared and annotated the datasets according to the workflows provided with each software documentation (manual, tutorial and readme available in Bioconductor or github repositories).

- *scType*: The standard approach requires the load of a database with predetermined positive and negative marker genes for each cell type. For each cell, *scType* returns a vector of scores that assigns the most frequently occurring cell type to the computed cell clusters. We first used *scType* for an overview of *adverSCarial* functioning with the PBMC3k *undivided set*. For further evaluation and comparison with the other classifiers, *training* and *test sets* were used.
- *CHETAH*: The default workflow implies manipulations of *SingleCellExperiment* (Amezquita *et al.* 2020) objects. Within *adverSCarial*, the *CHETAHClassifier* function inputs two *SingleCellExperiment* objects, one for annotating the *training set* as reference, and the other one for the *test set*. *CHETAH* confidence threshold parameter (*tresh*) was set to 0.01. For each cell, this classifier returns a predicted cell type and a score.
- *scAnnotatR*: The tool constructs an intermediate binary classifier based on an SVM algorithm for every cell type. Each of these intermediate models predicts the likelihood of a cell to belong to its respective type. The predictions are then integrated into a single model that outputs a vector of scores representing the probability of each cell type for each individual cell. The SVM classification necessitates a predefined list of genes to discriminate efficiently between cell clusters. Within *adverSCarial*, the *getSignGenes* function ranks the most significant genes between every 1 versus 1 cluster combination. Here we returned the top 20 of this ranked list as input genes for *scAnnotatR.*

*scRF* and *scMLP* were specifically implemented in the present work for the purpose of *adverSCarial* analysis. We built the models according to RF and MLP architectures, respectively, and trained them first with *balanced training sets*:

- *scRF*: This RF-based classifier was implemented with the *randomForest* package (version 4.7.1.1). The model was built with default parameters: the number of trees (*ntree*) was set to 500, and the number of variables randomly selected at each subdivision (*mtry*) was calculated as the square root of the number of predictor variables, which in PBMC3k was 117, in AXILLA10k was 159, in KIDNEY10k was 181, and in LIVER10k was 160. The response variable *y* was one-hot encoded to represent the cell types as a binary matrix, suitable for multinomial classification.
- *scMLP*: This MLP-based classifier was built with the R *Keras* library (version 2.15.0). The model architecture consisted of five fully connected layers. The first layer included 128 units with ReLU activation, followed by a dropout layer at a rate of 0.5 to prevent overfitting, then a layer of 64 units with ReLU activation, followed by another dropout layer at a rate of 0.5. The output layer consisted of units equal to the number of unique cell types in the dataset, using *softmax* activation function to normalize the output for multinomial classification over the cell types. The categorical *cross-entropy* loss function was used to measure the performance of the model. The adaptive moment estimation (*Adam*) optimizer (Kingma *et al.* 2017) was used for gradient descent computation during model updates. Training was performed with 20 epochs, a batch size of 32, and 20% of the data as validation.

### 2.3 Visualization

UMAP (Unified Manifold Approximation and Projection) plots were computed and rendered with *Seurat* (v5.0.3) by using the 2000 most variable genes of the dataset along with the genes of interest selected by the adversarial attacks (e.g. with *scType*, 2000 + 27 genes for *max-change*/*perc99*). Heatmaps were generated with *ComplexHeatmap* (v2.17) (Gu 2022) after hierarchical clustering on median-centered data with Euclidean distance and complete linkage. Each time, the clustering was performed on the 100 most discriminant genes (obtained with the *getSignGenes* function) along with the genes of interest selected by the adversarial attacks (e.g. with *scType*, 100 + the 4 genes *ICAM1*, *RPS27*, *ACTB* and *CAMKK2* for *single-gene*/*perc99*). Otherwise, graphics were generated with ggplot2 (v3.4.4).

## 3 Results

### 3.1 Implementation and algorithm

**Figure 1.**
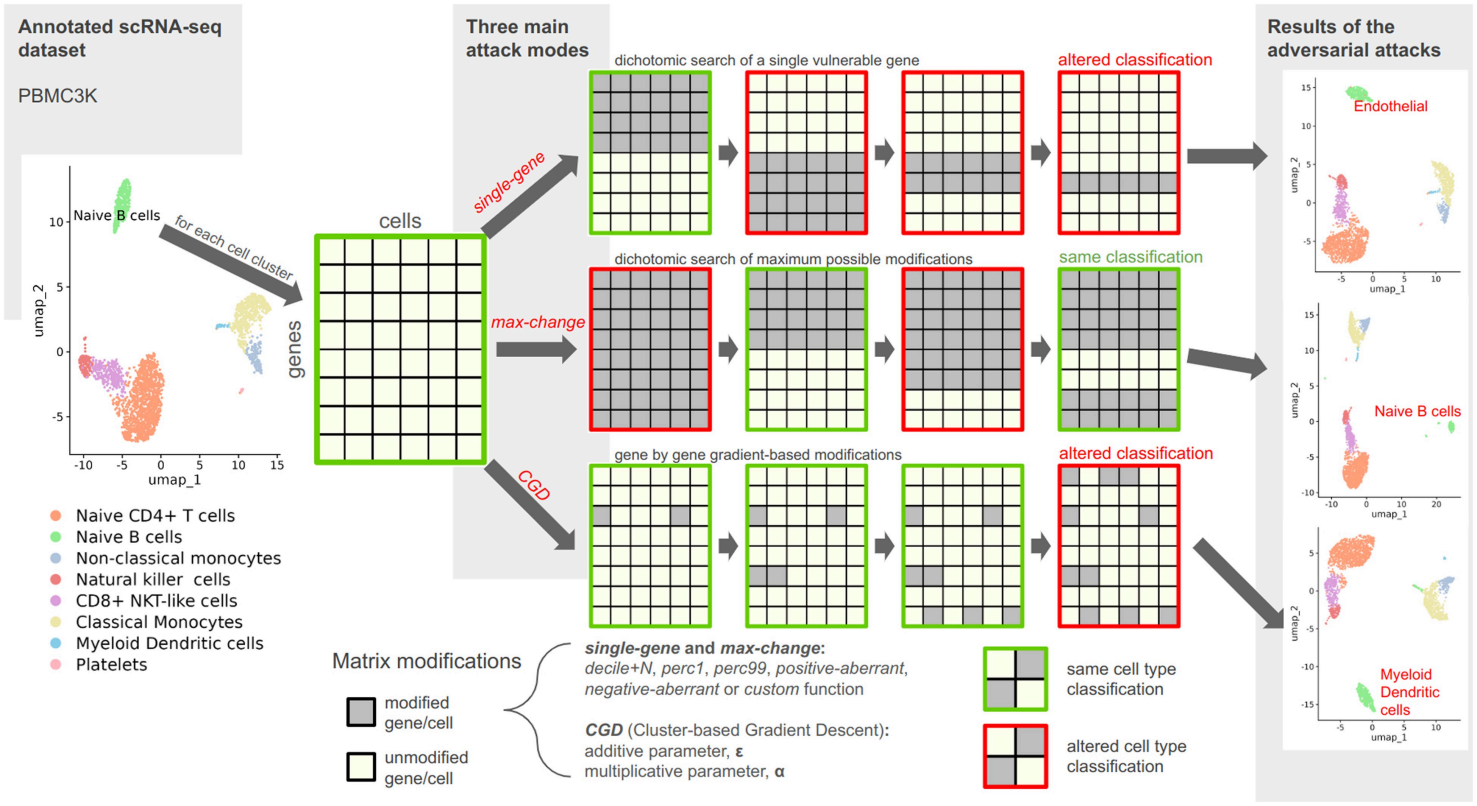
adverSCarial feature overview. Running of the *single-gene*, *max-change* and *CGD* (Cluster-based Gradient Descent) attack algorithms. UMAP representations of the *scType*-annotated PBMC3k dataset before and after *single-gene*, *max-change* or *CGD* attacks on the Naive B-cell cluster.

To be compatible with *adverSCarial*, classifiers should be set up as a function that inputs a gene × cell expression matrix, a categorical vector/list labeling each cell with the examined classifier, and the target label for the group of cells (cluster) under investigation (Fig. 1). The function should return the predicted cluster classification with its likelihood score, and a cell likelihood score × cell type matrix along with the predicted classification for each individual cell. This step ensures input/output encapsulation of any analysis mode, whether cell-wise or cluster-wise, into *adverSCarial*. Examples of preparation with *scType* (Ianevski *et al.* 2022) and *CHETAH* (de Kanter *et al.* 2019) as respective classifiers are provided in the Supplementary Material (Supplementary Data S1).

#### 3.1.1 Types of modifications

The different attack functions provided by the package require another critical parameter defining the gene modification method to be applied. Several gene expression modulations are available, reflecting a variety of biological, technical, or malicious events. Two modifications, ***perc1*** and ***perc99***, simulate an inactivation and activation by replacing all cell expression values for a given gene by the 1st and the 99th percentile, respectively. Genes or groups of genes can also be down- or upregulated in a synchronized manner using the ***decile-N*** or the ***decile+N*** modifications, which simulate a gradual decrease or increase by deciles of expression, on a scale *N*, with *N *=* *1 to 10. The ***random*** modification introduces uniform random noise between the minimum and the maximum expression values and simulates either real-world variations or errors in gene expression. With the ***custom*** function, users can also implement and apply any custom modification. For example, one can simulate the dropout effect frequently observed in single-cell transcriptomics and use the function to generate an attack (Supplementary Data S2). Last, we simulate potential technical errors or data processing miscalculations by introducing aberrant values. ***Positive-*** and ***negative-aberrant*** parameters create extreme outlier values by modifying gene expression levels with plus and minus 105 times the dataset maximum, respectively.

#### 3.1.2 scRNA-seq attack modes

- *Single-gene*: The *single-gene* attack produces cell misclassification and mislabeling after altering the expression of one gene only. The *advSingleGene* function is designed to identify every *single-gene* attack on a cell cluster given a modification type. On a computer with average technical specifications, most classifiers require >10 s to cluster a group of cells. As standard scRNA-seq datasets contain approximately 20 000 genes, a brute-force approach testing each gene individually would run for 55 h and be computationally intensive. Instead, the function uses a two-step strategy that first partitions the gene list into 100 bins (default settings), then applies a recursive dichotomous search into each bin to identify attackable genes, and finally isolates a single gene responsible for the misclassification (Fig. 1, Supplementary Data S3). The search for an item within an n-item list can be executed in *log2(n)* time: if the dataset contained a *single-gene* attack, it would necessitate 100 + *log2(20* *000)* = 114 runs and <20 min of computational time.
- *Max-change*: This attack (*advMaxChange* function) tries to modify as many genes as possible without affecting the cell clustering output and returns the complementary gene set: the genes that cannot be touched lest a misclassification occurs. Again, a brute force approach would quickly reach a complexity barrier [for an n-gene dataset: *O(2n)*]. Instead, the method alters the cell cluster on all genes and then splits the genes in two subsets when the classification changes. If further subset modification leaves the classification unchanged, it is combined with a previous classification-unchanging gene-set picked out in earlier stages, otherwise the subdivision continues. This process runs recursively until the largest unchanging set is found. The final complementary gene list consisting of the untouchable genes is referred to as the cell cluster signature (Fig. 1, Supplementary Data S4).
- *Cluster-based Gradient Descent (CGD): CGD* is implemented in the *advCGD* function and is a new gradient-derived adversarial perturbation method devised for the purpose of *adverSCarial* in-depth testing of black-box scRNA-seq classifiers. This iterative, cluster-based modification process (one gene by step, whole cluster perturbation instead of cells) is guided by an approximated gradient of the cell type likelihood with respect to a gene *j* expression. With the entire cell × gene matrix defined as *X*, the subset matrix corresponding to a cluster of cells *m* as *X_clust m_*, and the gene expression vector of a cell *i* as *x_i_*:
  $$\begin{array}{c} X={\left(x_{i}\right)}_{i\;\leq \;∥\mathrm{Cells}∥},\;\mathrm{and}\\ \begin{array}{c} x_{i}={\left(x_{ij}\right)}_{j\;\leq \;∥\mathrm{Genes}∥},\\ \begin{array}{c} {\hat{y}}_{x_{i}}={\left(score_{k}\right)}_{k\;\in \mathrm{CellTypes}}\;\mathrm{classifies}\;a\;\mathrm{cell}\;i,\;\mathrm{and}\\ {\hat{y}}_{X_{\mathrm{clust}\;m}}=\sum_{i\in \mathrm{clust}\;m}^{\;}e_{k^{\prime }}\;\mathrm{classifies}\;a\;\mathrm{cluster}\;\mathrm{of}\;\mathrm{cells}\;m\;\mathrm{with}\\ \;k^{\prime }\;≔\arg{\underset{k}{\max}\left({\hat{y}}_{x_{i}}\right)}_{k}. \end{array} \end{array} \end{array}$$
  Initially, the method infers the gradient of $\hat{y}\;$for each cell of *X_clust m_* with respect to a gene *j’* expression, by finite differences. The magnitude of this difference is adjusted by the two parameters *α* and *ε* (Fig. 1):
  (3) $$\begin{array}{c} \nabla_{j^{\prime }}^{fd}{\hat{y}}_{x_{i}}={\hat{y}}_{f_{j^{\prime }}\left(x_{i},\alpha ,\epsilon \right)}-{\hat{y}}_{x_{i}},\;\text{with}\\ f_{j\prime }\left(x_{i},\alpha ,\epsilon \right)=x_{ij}\left(1-\delta_{jj\prime }\right)+\left(\left(1+\alpha .\mathrm{sign}\left(x_{ij}\right)\right)x_{ij}+\epsilon \right)\delta_{jj\prime } \end{array}$$
  From both classifier results (by cell and by cluster), we define $\mathrm{ctype}1_{x_{i}}$ and $\mathrm{ctype}2_{x_{i}}$, the most and second most likely cell types of *xi*, and $\mathrm{ctype}1_{X_{\mathrm{clust}\;m}}\;$and $\mathrm{ctype}2_{X_{\mathrm{clust}\;m}}$, the most and second most likely cell types of *X_clust m_*, respectively. *CGD* follows the inferred gradient to lower the gap between $\mathrm{ctype}1_{X_{\mathrm{clust}\;m}}$ and $\mathrm{ctype}2_{X_{\mathrm{clust}\;m}}$ scores by modifying the genes on each cell of *m* one by one, until $\mathrm{ctype}2_{X_{\mathrm{clust}\;m}}$ becomes the new most likely cell type predicted by the classifier. The adversarial matrix $X_{\mathrm{clust}\;m}^{\mathrm{adv}}$ is returned, with the step size, and thus the attack level, set by the same multiplicative and additive parameters *α* and *ε*, respectively:
  (4) $$\begin{array}{c} s\mathrm{lope}={\left(\nabla_{j\prime }^{fd}{\hat{y}}_{x_{i}}\right)}_{\mathrm{ctype}2{}_{X_{\mathrm{clust}\;m}^{n}}}-{\left(\nabla_{j\prime }^{fd}{\hat{y}}_{x_{i}}\right)}_{\mathrm{ctype}1{}_{X_{\mathrm{clust}\;m}^{n}}},\\ {\left(x_{i}^{n+1}\right)}_{j}=x_{ij}\left(1-\delta_{jj\prime }\right)+\left(\left(1+\alpha .\mathrm{sign}\left(x_{ij}\right)s\mathrm{ign}\left(\mathrm{slope}\right)\right)x_{ij}+\epsilon .\mathrm{sign}\left(\mathrm{slope}\right)\right)\delta_{jj\prime },\\ X_{\mathrm{clust}\;m}^{ad\upsilon }={\left(x_{i}^{n+1}\right)}_{i\;\in \mathrm{clust}\;m\;\;} \end{array}$$
  To reduce the number of iterations required for the adversarial attack, and because each step targets a different gene, we prioritize genes based on their likelihood of altering the classifier's output. The function *getSignGenes* can be used to rank the genes according to their inter-cluster differential t-statistics while ensuring their differentiating between all possible pairs of clusters (Supplementary Data S5). To avoid unnecessary computations, $x_{i}^{n+1}=x_{i}^{n}$ is set when $\mathrm{ctype}1_{x_{i}^{n}}=\mathrm{ctype}2_{X_{\mathrm{clust}\;m}^{n}}$ or *slope = 0* (the applied modification did not change the output of the classifier).
- *Random-walk*: This attack (*advRandWalkMinChange* function) applies various random modifications on a subset of genes and then progressively reduces this initial combination of genes × modifications while reclassification occurs. With this function, classifiers can be mined arbitrarily for fortuitous vulnerabilities and working attacks can be quickly identified.
- *Brute-force*: Last, the *brute/grid* attack (*advGridMinChange* function) exhausts every possible attack mode × modification type for a reduced number of starting genes. The combinatorial complexity of such an approach limits the number of genes to be tested to <10.

### 3.2 Showcase example of *adverSCarial* use

We performed an overall survey of the scRNA-seq marker-based classifier *scType* (Ianevski *et al.* 2022), applied on the PBMC3k *undivided set* (Supplementary Data S6). Using *adverSCarial* and the *single-gene* attack, we investigated whether modifying a single marker could alter the classifier output and to what extent. The *perc1* modification emerged as the least impactful with only a few potential attacks by cell cluster (mean 1, range 0–5). In contrast, *positive* and *negative-aberrant* modifications produced numerous misclassification events (mean 151 and 30.8, range 144–158 and 26–34, respectively), while *perc99* induced a moderate number of attacks (mean 11.9, range 0–40). The overall vulnerability to *single-gene* attacks was equally variable depending on the cell type, with platelet cells demonstrating the highest resistance and no weaknesses to the *perc1* or *perc99* modifications. Figure 1 (on the top-right corner) showcases the misclassification of the naive B cell cluster into endothelial cells after switching on *ICAM1* with *perc99* (more details in Supplementary Data S6). With the *max-change* attack, we further explored the minimum signatures of unalterable genes defining a correct classification with *scType*. Here the naive B cell cluster remained unaltered even after switching on 99.5% of all genes. The remaining 9 candidates thus constituted a critical gene set defining naive B cells for this classifier. Interestingly, there was an overall correlation between the reported signature lengths and the vulnerability to *single-gene* attacks with *scType*, after exclusion of the *random* modification method which by nature cannot be reproduced between analyses (Spearman’s rho = 0.66).

### 3.3 Evaluation of different types of scRNA-seq classifiers

We next conducted a comprehensive examination of the robustness of five distinct ML models dedicated to classifying scRNA-seq data. Three of these classifiers were sourced from existing literature: *scType* (marker-based model) (Ianevski *et al.* 2022), *CHETAH* (hierarchical classification) (de Kanter *et al.* 2019), and *scAnnotatR* (SVM) (Nguyen and Griss 2022). For the purpose of this evaluation, we implemented and trained two new classifiers, one based on random forests, hereby called *scRF*, and one deep learning neural network model based on a multilayer perceptron, called *scMLP*. Additionally, we tested four different datasets, PBMC3k, LIVER10k, AXILLA10k and KIDNEY10k, each divided into *training* and *test sets* (see Section 2).

#### 3.3.1 Survey of vulnerability to adversarial attacks

**Figure 2.**
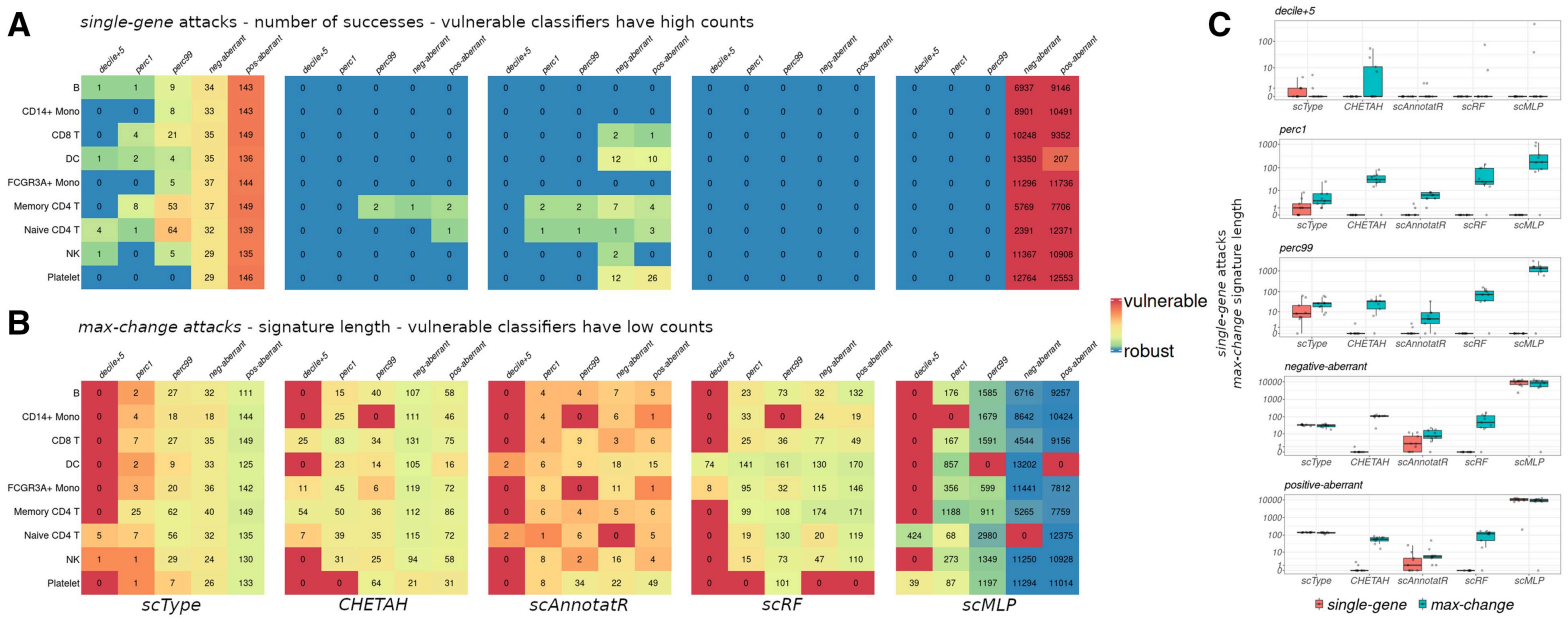
Vulnerabilities of five scRNA-seq classifiers to *single-gene* and *max-change* adversarial attacks. After a training step on the PBMC3k training set, five modification types are sequentially applied on the nine cell types of the test set. Heatmaps reporting (A) the number of successful *single-gene* attacks (lower is better), and (B) the signature length of *max-change* attacks (higher is better) by classifier, cell type and modification type. For *single-gene*, counts > 0 point out vulnerabilities, while classifiers are considered robust to *max-change* attacks with high counts. (C) Boxplots summarizing the impact of each modification (sum over each cell type) for both attack modes, by classifier. Data are represented in log10 scale.

*Single-gene* attacks on the five classifiers revealed varying susceptibilities depending on the class of algorithm tested, ranging from *scType* often being the most vulnerable, to *scRF* being entirely impervious to all modification types (Fig. 2A). With this attack mode, *decile + 5* was the least effective modification and solely affected *scType*, with seven possible attacks (Supplementary Data S7). *Perc1* and *perc99* modifications were effective on *scType*, *CHETAH*, and *scAnnotatR* with 185, 2, and 6 successful attacks, respectively (Supplementary Data S8), while *positive-* and *negative-aberrant* modifications yielded >1500 results for *scType*, 4 for *CHETAH*, 80 for *scAnnotatR*, and about 200 000 for *scMLP*. Concerning *max-change* attacks, the same trends were observed for all classifiers, from *decile + 5* producing the shortest signature lengths to *positive-* and *negative-aberrant* modifications yielding the longest ones (Fig. 2B). Dependent of a classifier/modification combination, situations arose for every classifier where all genes of all cells from a cluster could be modified without changing the prediction (signature length = 0), including the extreme *positive-* or *negative-aberrant* modifications. Overall, relatively to each other and given the extent of the presented modifications with all classifiers, *decile + 5* was a low-impacting modification while *perc1* and *perc99* were medium-impacting, and *positive-* and *negative-aberrant* were high-impacting for both attack modes (Fig. 2C). Both classifiers *CHETAH* and *scRF* showed few responses to medium or high modifications, indicating a plateau in sensitivity due to a natural characteristic of tree-based algorithms: once an input reaches the defined threshold of a tree node, further increases have no additional impact on the outcome (Tan *et al.* 2018). In contrast, *scMLP* was unresponsive below medium modifications and very vulnerable beyond with thousands of possible attacks: this effect stems from the neural network's use of multiplicative coefficients, which amplify sensitivity to outliers (Tan *et al.* 2018).

#### 3.3.2 Detectability of the modifications

**Figure 3.**
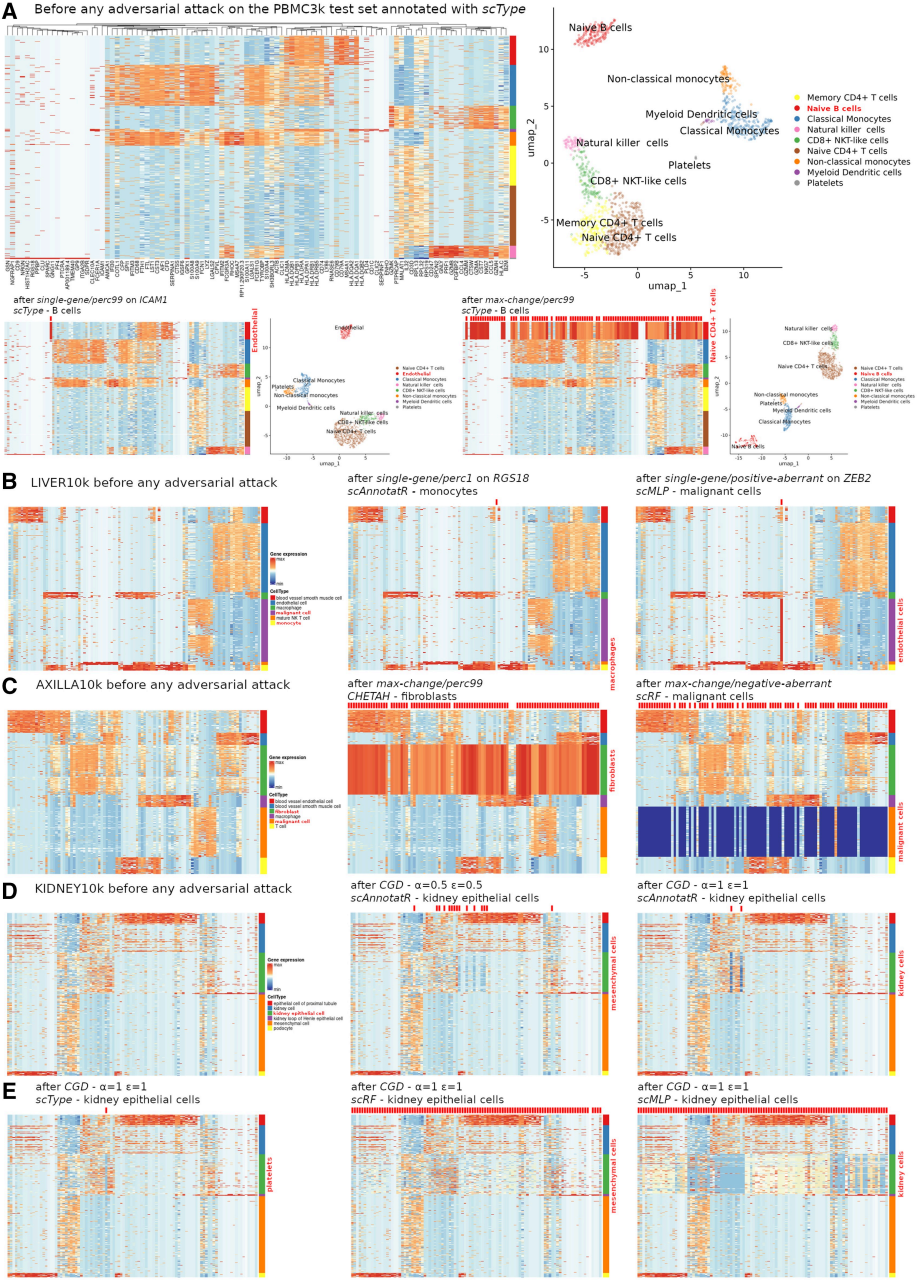
Perceptibility of the changes induced by various adversarial attacks. Classifiers are trained and tested with training and test sets, respectively. On top of each heatmap, applied modifications are marked with discrete ticks, whether visible or not. (A) Hierarchical clustering heatmaps and UMAP representations after classification and annotation with *scType*, before (on top) and after a *single-gene* attack (bottom-left) or a *max-change* attack (bottom-right) on PBMC3k B cells. (B) Hierarchical clustering heatmaps of the most discriminating genes before and after various *single-gene* attacks of different scRNA-seq classifiers (dataset: LIVER10k). (C) Idem after various max-change attacks (dataset: AXILLA10k). (D) Idem after *CGD* attacks of incrementing magnitudes (parameters *α* and *ε*) on scAnnotatR (KIDNEY10k). (E) Idem after *CGD* attacks of comparable levels on different classifiers (KIDNEY10k).

An essential aspect of adversarial attacks is the human perceptibility of the introduced changes (Goodfellow *et al.* 2015). Confirming the results obtained with *scType* on the PBMC3k *undivided set*, a *single-gene* and *max-change*/*perc99* attacks on B cells from the *test set* revealed critical features for the classification, *ICAM1* and a 27-gene signature, respectively, while maintaining a comparable cell cluster topology on UMAP (Fig. 3A). Of note, clustering heatmaps enhance visual detectability of these features along with the applied modifications by focusing on the 100 most variable genes discriminating between each cell cluster instead of displaying all genes (see Section 2). On other datasets, *single-gene* attacks produced variably discernible alterations in the targeted gene’s expression but preserved the visual patterns of the cell type (Fig. 3B). Expectedly, *max-change* attacks with *perc99* and *perc1* obliterated the visual patterns of the targeted cell clusters but did not alter the classification (Fig. 3C). For *CHETAH* and *scRF*, the respective residual signatures contained 793 and 4506 genes. Allowing for control over modification intensity using *α* and *ε* parameters, the *CGD* algorithm yielded subtle modifications with low values such as *α* = *ε* = 0.5 to more visible ones with *α* = *ε* = 1. However, with low values the attacks had to impact many genes to fool the classifier, whereas far less genes were needed with higher values. For example, *CGD* slightly adjusted 15 genes to alter *scAnnotatR* classification of the kidney epithelial cell cluster, but only 2 genes had to be ostensibly modified for the same result (Fig. 3D). Depending on the classifier, equal parameter values produced different results, from 1 gene for *scType* to 577 genes for *scMLP* with *α* = *ε* = 1 (Fig. 3D and E).

#### 3.3.3 Performance, accuracy, and prediction dynamics

**Figure 4.**
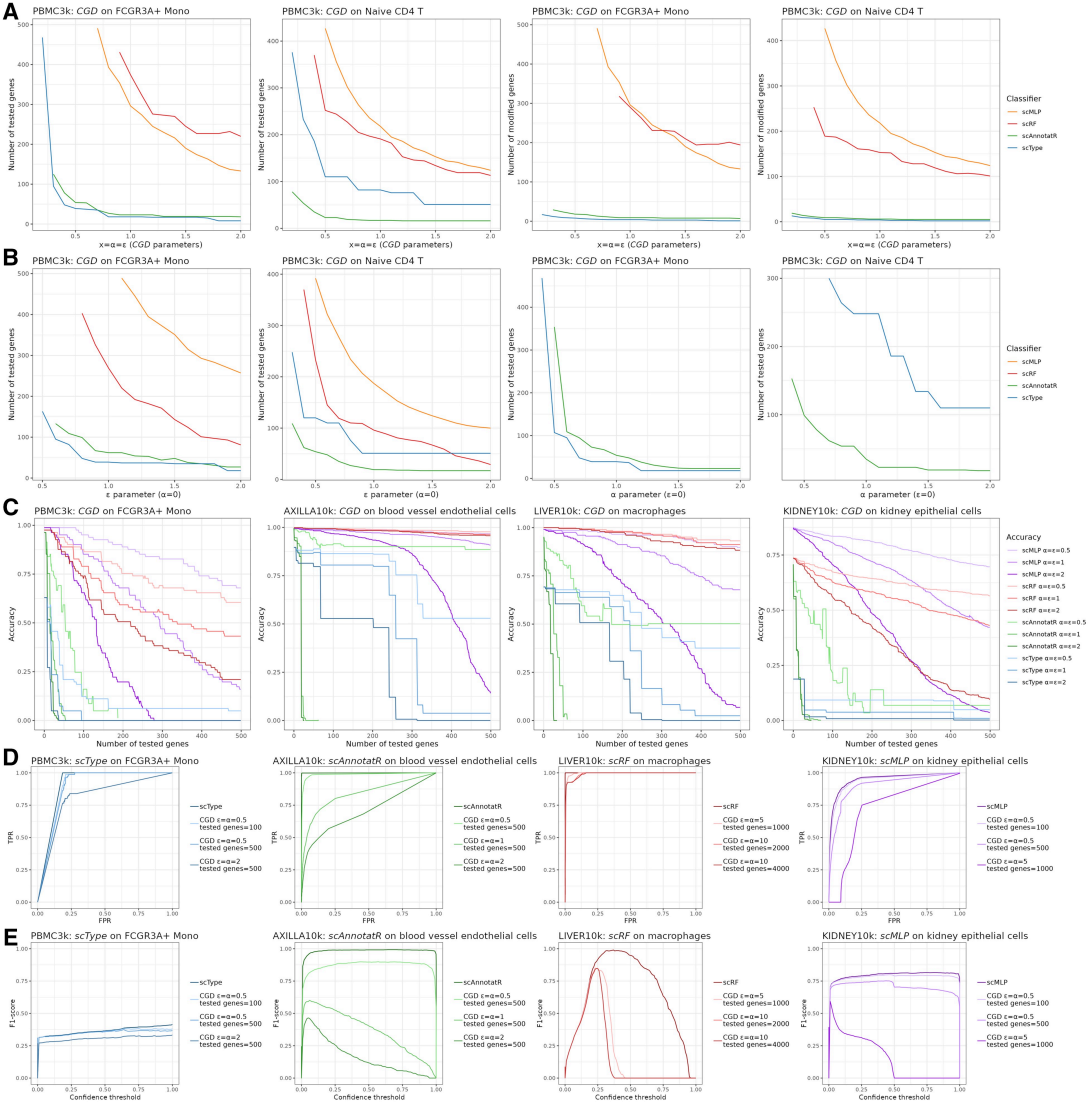
Performance and evaluation of the cluster-based gradient descent (*CGD*) algorithm. *CGD* has been applied to the four eligible classifiers *scType*, *scAnnotatR*, *scRF*, and *scMLP*. (A) Number of genes to be tested and/or modified for deviating the classification of the cell cluster, according to equal parameters *α* and *ε*. (B) Idem according to *ε* when *α* = 0 (left), and to *α* when *ε* = 0 (right). (C) Accuracy of the prediction according to the number of tested genes, with different sets of values for *ε* and *α*, on different datasets. (D) ROC curves and (E) corresponding F1-scores for different values of *ε*, *α* and the number of tested genes, on different datasets. FPR: false positive rate, TPR: true positive rate.

A notable feature of the *CGD* algorithm is that it modifies only some of the genes and stops after the cell cluster’s classification has switched. Thus, only the genes influencing the output are modified, which can lead to a discrepancy between the number of genes tested and those modified. The ratio of tested genes/modified genes differed among the eligible classifiers for *CGD* adversarial attacks (Fig. 4A). For instance, on the PBMC3k FCGR3A+ Mono cell cluster, an attack of *scType* with *α* = *ε* = 1 required testing 18 genes but modified only 4 genes (0.22-fold). For *scAnnotatR*, there was 23 tested/9 modified genes (0.39-fold) with the same parameters. With both classifiers, this gap can be explained by the underlying algorithms’ architectures, one being marker-based and the other SVM-based: there is a selection of input variables for classification. Conversely, *scRF* tested 374 genes and modified 290 of them (0.78-fold) while *scMLP* tested 296 genes and modified them all. These two models inherently consider nearly all input variables, even if their contribution to the prediction is marginal. Between these two groups of classifiers (selective versus unselective in their inputs), responses were also different when testing the influence of the parameters *α* and *ε* independently (Fig. 4B). Each classifier could be attacked solely with the *ε* additive part of the algorithm. However, with a maximum of 500 tested genes, *scMLP* and *scRF* could not be attacked by exclusively using the *α* multiplicative part. To enable accuracy and prediction dynamics’ analyses as functions of the number of tested genes, we implemented an augmented version of *CGD* that continues the iterative process until all provided genes are tested, even after the cell cluster predictions are deviated from the original cell type. As compared to *scRF* and *scMLP*, *scType* and *scAnnotatR* exhibited disturbances more rapidly and switched predictions with fewer genes. For each classifier, higher values of *α* and *ε* were associated with greater disturbances (Fig. 4C). ROC curves varied depending on the classifier and the targeted cluster. For *scAnnotatR*, performance was significantly impacted by attacks with standard parameters *α* = *ε* = 1 and 500 tested genes. In contrast, *scRF* showed only minor performance changes even with high parameter values such as *α* = *ε* = 10 and 4000 tested genes (Fig. 4D). In the same conditions, the F1-score analysis revealed that the classifiers were primarily affected when a high probability threshold was set for predicting a positive (Fig. 4E).

### 3.4 Which attack/modification for my classifier/data?

Upon selecting an attack method, several factors need to be considered to evaluate the success rate and computation time required with *adverSCarial* (Table 1):

**Table 1.** Examples of *adverSCarial* use on different datasets and classifiers, along with parameters, outcomes, computational resources, and specifications.

Targeted classifier	Targeted dataset				
	PBMC3k	AXILLA10k	KIDNEY10k	LIVER10k	
Adversarial attacks: *single-gene* with *perc1* modifications					

*scType*	NK	Malignant cells	Kidney epithelial cells	Blood vessel smooth muscle cells	Targeted cluster
	72	1627	1325	505	No. of cells
	0	0	2	1	No. of successful attacks
	4 min	2 h	4 h	3 h	Runtime
	Intel i7, RAM 8 GB				Specifications

Adversarial attacks: *max-change* with *decile + 5* modifications, *MaxSplitSize *=* *1000					

*scRF*	memory CD4 T	Blood vessel endothelial cells	Podocytes	Malignant cells	Targeted cluster
	172	744	139	1917	No. of cells
	0	0	7183	0	No. of signature length
	3 min	20 min	9 h	40 min	Runtime
	Intel i7, RAM 8 GB				Specifications

Adversarial attacks: *max-change* with *perc99* modifications, *MaxSplitSize *=* *50					

*CHETAH*	memory CD4 T	Blood vessel endothelial cells	Podocytes	Malignant cells	Targeted cluster
	172	744	139	1917	No. of cells
	941	546	448	0	No. of signature length
	2 h	2 h	16 h	3 min	Runtime
	Intel i5, RAM 64 Gb				Specifications

Adversarial attacks: *CGD* with *α* = ε = 0.5					

*scAnnotatR*	CD8 T	Blood vessel smooth muscle cells	Mesenchymal cells	Macrophages	Targeted cluster
	139	389	2559	205	No. of cells
	memory CD4 T	Fibroblast	Kidney cell	Malignant cell	New cell type
	10	17	20	28	No. of modified genes
	2 min	30 min	1 h	56 min	Runtime
	Intel i5, RAM 64 Gb				Specifications

*scMLP*	CD8 T	Blood vessel smooth muscle cells	Mesenchymal cells	Macrophages	Targeted cluster
	139	389	2559	205	No. of cells
	NK	Fibroblast	Kidney epithelial cell	Monocyte	New cell type
	151	2121	1109	1806	No. of modified genes
	4 min	5 h	4 h	4 h	Runtime
	Intel i5, RAM 64 Gb				Specifications

Adversarial attacks: *CGD* with *α* = ε = 10					

*scMLP*	CD8 T	Blood vessel smooth muscle cells	Mesenchymal cells	Macrophages	Targeted cluster
	139	389	2559	205	No. of cells
	NK	Fibroblast	Kidney epithelial cell	Monocyte	New cell type
	3	43	55	65	No. of modified genes
	6 s	6 min	11 min	9 min	Runtime
	Intel i5, RAM 64 GB				Specifications

- *Computing resources*: Each step of the three main attack modes involves modifying a matrix and classifying the result to evaluate their impact. The modifications applied to one gene take an average of 9 s with an Intel(R) Core(TM) i7-8550U (base clock speed: 1.80 GHz) with 8 Gb of DDR4 RAM and 1 s with an Intel(R) Core(TM) i5-7400 (base clock speed: 3.00GHz) with 64 Gb of DDR4 RAM (Supplementary Data S9). The computation time also depends on the classifier, averaging on 2 min for *scMLP* to 9 min for *scAnnotatR* with 8 Gb RAM, and 1 min for *scMLP* to 5 min for *scAnnotatR* with 64 Gb RAM (Supplementary Data S10).
- *Single-gene*: With no attack possible, the algorithm evaluates each of the initial 100 gene bin once and returns an empty result. However, with multiple possible attacks, a binary search is initiated within each potential bin, increasing the computational time (Supplementary Data S11). For example, an unsuccessful attack on an AXILLA10k cluster with *scType* consumed 2 h of runtime as compared to 4 h for a successful one on a KIDNEY10k cluster containing less cells (Table 1).
- *Max-change*: When the recursive process involves low-impacting modifications that are less likely to alter the classification, a substantial number of genes can be integrated into the search bin early on, sometimes encompassing the entire set (as indicated by a signature length of 0). In contrast, high-impacting modifications require deeper recursive searches to identify gene sets associated with no classification change and require >2 h of runtime if successful. For faster computation, the *maxSplitSize* option can be used to stop the recursion once a specified gene bin size bas been reached. Setting higher values for *maxSplitSize* reduces the computational resources but increases the size of the identified gene set, resulting in a less specific signature.
- *CDG*: With CGD, computation time primarily depends on *α* and *ε* as higher values for these parameters reduce the number of genes that need to be modified to achieve a successful attack. For instance, with *scMLP* and *α* = *ε* = 0.5, 3 h of runtime and 1297 modified genes are required, which is contrasted by 7 min of runtime and 42 modified genes with *α* = *ε* = 10.

*Overview mode*: Finally, *adverSCarial* implements tools for the assessment of overall attack susceptibilities in a complete dataset given a classifier (Supplementary Data S12). The user can quickly review both *single-gene* and *max-change* attacks in combination with various modification modes to gain insights on what works best for a specific classifier/dataset pair. Given the time intensive nature of testing every cell cluster across multiple modifications, a less precise yet faster configuration of these attack modes is used. First, recursive subdivisions are halted once the subsets reach 100 genes; second, the *single-gene* mode begins with fewer initial bins by default (20 subsets). These approximations allow for a quick overview of potentially hazardous modification types and point out cell clusters for any kind of vulnerability. If needed, precise attack runs can next be set up with this information.

## 4 Discussion

Through this examination of five scRNA-seq classifiers on four different datasets, we unveiled a spectrum of weaknesses to adversarial attacks dependent of the underlying class of ML algorithm and the extent of the applied modulations. For instance, we provide a clear demonstration that outliers and aberrant values easily deviate the tools’ predictions and need to be filtered in preprocessing steps as a prerequisite for correct operation. This, however, might not be sufficient for flawless reports in cases of slight technical or even biological biases. Eventually, every classifier failed after a threshold of accumulated modifications was reached: this threshold was one of the most constant characteristics observed during the course of this survey. From substantial changes applied on a few genes only to invisible ones applied on hundreds of genes, susceptibilities arose preferentially with each class of models, but the sum of modifications did not differ drastically. Accuracy analyses, as well as ROC and F1-score curves further revealed differences in classifier behavior following diverse attacks, highlighting general vulnerabilities and the specific ways with which each algorithm respond. Similar to what has been observed in other fields, notably in image recognition, none of the ML models examined here were impervious to discrete and imperceptible changes throughout the information matrix (Goodfellow *et al.* 2015, Han *et al.* 2020, Zhou *et al.* 2021, Ghaffari Laleh *et al.* 2022).

As compared to existing adversarial tools, *adverSCarial* presents the advantage of being compatible with various classifier algorithms, potentially all types of models working with any kind of scRNA-seq data. It offers distinct adversarial attack modes, two that do not require access to the gradient and another one that approximates it, enabling testing on nearly any white-box and black-box ML model. By handling *Seurat* or *SingleCellExperiment* objects directly, by encapsulating the classifier’s functioning and parameters, *adverSCarial* ensures minimal data manipulation from the beginning, simplifying the user’s experience. Future versions could include parallel processing to reduce computational time and incorporate other adversarial algorithms, such as FGSM and PGD, to enable broader analyses. For example, several versions and improvements of the *CGD* algorithm could be explored. A simpler algorithm disregarding the likelihood of the second most likely cell type would be useful for classic adversarial training tasks, such as improving a classifier’s robustness or accuracy. Moreover, *CGD* modifies a gene whenever it exerts an influence, however marginal, on the classifier's outcome. With deep learning models this process might not always be efficient because the predicted outputs are based on nearly every input variable. Introducing a gradient threshold for modifying a gene could enhance efficiency by ensuring that only relevant changes are implemented from the start. Another avenue for improvement lies in the preselection of genes to be modified. In this work, we first and foremost aimed to evaluate a diversity of classifier types, and to gain insights on performance, accuracy and prediction dynamics during attacks with *adverSCarial*. Therefore, we prioritized the genes to be tested by preliminary rankings with differential statistics between all possible cell types. This is not ideal in adversarial attacks where targeting less conspicuous markers might be preferable, e.g. to leverage *CGD*’s potential for marker discovery. To address this limitation, and because testing every single gene with a gradient-based algorithm is resource-consuming, alternative gene preselection strategies could be considered, whether at random, according to the variance observed, or by establishing scores reflecting phenotypic features.

Considering that classic adversarial attack analyses primarily aim to evaluate the robustness of systems against various forms of input variability, whether malicious or not, certain observations still suggested a potential for cell marker differentiation using these methods (Supplementary Data S13). The present survey highlighted several genes during *single-gene* or *max-change* attacks: on B cells, switching *ICAM1* on changed *scType’*s prediction to endothelial cells; on naive CD4 T cells, switching *RPS27* off changed *scAnnotatR*’s prediction to memory CD4 T cells; *CHETAH* and *scMLP* were also fooled by modifications on *ACTB* (from memory to naive CD4 T cells) and *CAMKK2* (from CD8 T to FCGR3A+ Mono cells), respectively. If *RPS27* and *ACTB* were effectively differentially expressed in naive versus memory CD4 T cells (adjusted *P*-values = 1.69 × 10^−5^ and 3.06 × 10^−9^, respectively), hence discoverable with usual approaches, in the case of *CAMKK2* differential statistics in CD8 T versus FCGR3A+ Mono cells were not significant (adjusted *P*-value = 0.21). As another example, the CD14+ monocyte signature included several statistically significant ribosomal genes, such as RPL13A, RPL21, RPS15A, RPS23, RPS27A, RPS4X, RPS4Y1, and RPS5, and others that were not: RPL29 and RPS11. Thus, among the genes identified through adversarial attacks that do not overlap with those reported by standard explorations discriminating between cell types, such as statistical analyses, dimension reduction and clustering techniques, some candidates could be informative to expose the inner workings of the ML models. When for instance, the modification of a single gene typically should not suffice to change the prediction of a marker-based classifier. Nevertheless, whenever an original signature is uncovered, cross-validations by redundancy should be endeavored and gene overlaps estimated (Cantini *et al.* 2018, Chi *et al.* 2020).

In conclusion, *adverSCarial* introduces novel approaches to pinpoint weaknesses against adversarial attacks and evaluate the robustness of single-cell-transcriptomics classifiers. The diverse functionalities allow flexible modulations in attack modes and modification types, ranging from subtle and virtually undetectable changes to clearly visible and aggressive alterations. On hallmark scRNA-seq datasets and ML tools such as *scType, CHETAH*, *scAnnotatR*, and specifically implemented RF and MLP-based models, it highlighted many vulnerabilities and susceptibility profiles to gene expression modifications, underscoring the remaining challenges left to ensure accurate classification following technical, experimental design, and malicious events. As single-cell transcriptomics continuously expands in many domains, including clinical use and precision medicine, we believe *adverSCarial* can be a useful toolkit for researchers, developers and end-users to survey the vulnerability, address security concerns, and identify the inner decision-making mechanisms of the ML algorithms exploiting scRNA-seq data.

## Supplementary Material

btaf168_Supplementary_Data

## Data Availability

The data and code underlying this article are available in GitHub at https://github.com/GhislainFievet/adverSCarial (main development branch) and https://github.com/GhislainFievet/adverSCarial_article (supplementary code and data) and can be accessed freely.
