# Supplementary material for: adverSCarial: assessing the vulnerability of single-cell RNA-sequencing classifiers to adversarial attacks
Source: Bioinformatics. 2025 Apr 15;41(4):btaf168. doi: 10.1093/bioinformatics/btaf168 (PMC12036967; doi:10.1093/bioinformatics/btaf168)
Supplement: btaf168_Supplementary_Data [file btaf168_supplementary_data.pdf]

Supplementary Data A

Prepare a classifier with `CHETAH` and `scType`

All classifiers needs to be formated in a certain way to be compatible with the adverSCarial package. We provide an example on a simple formating with `CHETAH`, and a more advanced formating with `scType`.

`CHETAH`

Here we demonstrate how to implement a classifier working with the single-gene and max-change attack, by taking the example of `CHETAH` a Bioconductor scRNA-seq classifier.

Load data

```
library(adverSCarial)
library(TENxPBMCData)
library(CHETAH)
library(scater)
library(scran)
```

First let’s load a `train` and a `test` dataset.

```
train_3k <- TENxPBMCData(dataset = "pbmc3k")
test_4k <- TENxPBMCData(dataset = "pbmc4k")

cell_types_3k <- system.file("extdata", "pbmc3k_cell_types.tsv",
package="adverSCarial")
cell_types_3k <- read.table(cell_types_3k, sep="\t")
colData(train_3k)$celltypes <- cell_types_3k$cell_type
colnames(train_3k) = colData(train_3k)[['Barcode']]
colnames(test_4k) = colData(test_4k)[['Barcode']]
```

Then we process the `test_4k` to annotate and visualize the cell types.

We annotate cells with `CHETAH`.

```
input <- CHETAHclassifier(input = test_4k, ref_cells = train_3k)
input <- Classify(input = input, 0.00001)
colData(test_4k)$celltypes <- input$celltype_CHETAH
```

Process data.

```
test_4k <- logNormCounts(test_4k)
dec <- modelGeneVar(test_4k)
hvg <- getTopHVGs(dec, prop=0.1)
test_4k <- runPCA(test_4k, ncomponents=25, subset_row=hvg)
test_4k <- runUMAP(test_4k, dimred = 'PCA')
```

Visualize the results

```
plotUMAP(test_4k, colour_by="celltypes")
```

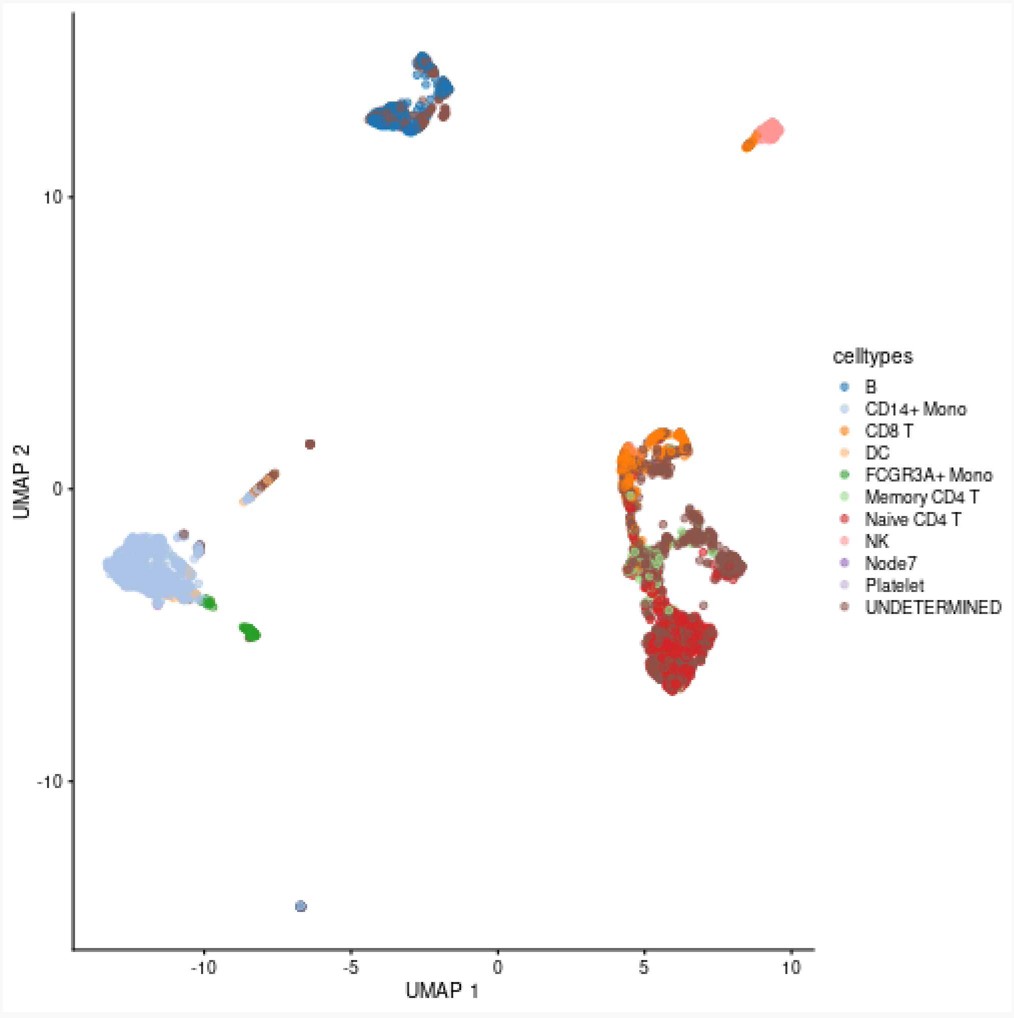

Adapt the classifier

`CHETAH` is a classifier that, when given a SingleCellExperiment object, returns a specific cell type from each cell. We need to adjust the classifier so that it can be used by *adverSCarial*.

Each classifier function has to be formated as follow to be used with the following functions: advSingleGene, advMaxChange, advGridMinChange, advRandWalkMinChange, maxChangeOverview, minSingleGeneOverview.

```
   classifier = function(expr, clusters, target){

                # `score` should be numeric between 0 and 1
                # 1 being the highest confidance into the cell type classification.
                c(
                    prediction="cell type",
                    odd=score)
    }
```

The `expr` argument contrains the RNA expression values, can be a *matrix*, a *data.frame* or a *SingleCellExperiment*. The list `clusters` consists of the cluster IDs for each cell in `expr`, and `target` is the ID of the cluster for which we want to have a classification. The function returns a vector with the classification result, and a trust indice.

This is how you can adapt `CHETAH` for `adverSCarial`.

```
CHETAHClassifier <- function(expr, clusters, target){
    if (!exists("reference_3k")) {
        reference_3k <<- train_3k
    }
    input <- CHETAHclassifier(input = expr, ref_cells = reference_3k)
    input <- Classify(input = input, 0.01)
    final_predictions = input$celltype_CHETAH[clusters == target]
    ratio <- as.numeric(sort(table(final_predictions), decreasing = TRUE)[1]) /
        sum(as.numeric(sort(table(final_predictions), decreasing = TRUE)))
    predicted_class <- names(sort(table(final_predictions), decreasing = TRUE)[1])
    if ( ratio < 0.3){
        predicted_class <- "NA"
    }
    c(prediction=predicted_class,
      odd=ratio)
}
```

This classifier takes as input a SingleCellExperiment object, you need to specify the `argForClassif="SingleCellExperiment"` argument in *adverSCarial* function. If your classifier takes as input a *matrix* or a *data.frame* you can let the default `argForClassif="data.frame"` argument.

You can now test `CHETAH` classifier with `adverSCarial` tools.

Let's run a `maxChangeAttack`.

```
adv_max_change <- advMaxChange(test_4k, colData(test_4k)$celltypes, "CD14+ Mono",
CHETAHClassifier, advMethod="perc99", maxSplitSize = 2000,
argForClassif="SingleCellExperiment")
```

Let's run this attack and verify if it is successful.

First we modify the `test_4k` SingleCellExperiment object on the target cluster, on the genes previously determined.

```
test_4k_adver <- advModifications(test_4k, adv_max_change@values,
colData(test_4k)$celltypes, "CD14+ Mono", argForClassif="SingleCellExperiment")
```

Then we verify that classification is still `DC`.

```
rf_result <- CHETAHClassifier(test_4k_adver, colData(test_4k)$celltypes, "CD14+
Mono")

rf_result

## [1] "CD14+ Mono" "1"
```

`scType`

Here we demonstrate how to implement a classifier working with all the attacks, include the gradient-based CGD, by taking the example of `scType` a scRNA-seq classifier.

Ianevski, A., Giri, A.K., Aittokallio, T. Fully-automated and ultra-fast cell-type identification using specific marker combinations from single-cell transcriptomic data. Nat Commun, 2022;13:1246. https://doi.org/10.1038/s41467-022-28803-w

Load data

```
df_pbmc_test <-
read.table("/home/gui/Dropbox/INSERM/jupyterlab/0033_these/data/v2/seurat_scaled_pbmc
_test.txt")
expr_df <- df_pbmc_test[, -which(names(df_pbmc_test) == "y")]
clusters_df <- df_pbmc_test$y
names(clusters_df) <- rownames(df_pbmc_test)
```

RNA expression matrix.

```
expr_df[1:5,1:5]
```

```
##                    AL627309.1  AP006222.2 RP11.206L10.2 RP11.206L10.9
## AAACATACAACCAC-1 -0.05812316 -0.03357571   -0.04166819   -0.03364562
## AAACATTGATCAGC-1 -0.05812316 -0.03357571   -0.04166819   -0.03364562
## AAACGCACTGGTAC-1 -0.05812316 -0.03357571   -0.04166819   -0.03364562
## AAATGTTGCCACAA-1 -0.05812316 -0.03357571   -0.04166819   -0.03364562
## AACACGTGCAGAGG-1 -0.05812316 -0.03357571   -0.04166819   -0.03364562
##                     LINC00115
## AAACATACAACCAC-1 -0.08223981
## AAACATTGATCAGC-1 -0.08223981
## AAACGCACTGGTAC-1 -0.08223981
## AAATGTTGCCACAA-1 -0.08223981
## AACACGTGCAGAGG-1 -0.08223981
```

Cell clusters

```
head(clusters_df)
```

```
## AAACATACAACCAC-1 AAACATTGATCAGC-1 AAACGCACTGGTAC-1 AAATGTTGCCACAA-1
##   "Memory CD4 T"   "Memory CD4 T"   "Memory CD4 T"   "Memory CD4 T"
## AACACGTGCAGAGG-1 AACCGCCTAGCGTT-1
##   "Memory CD4 T"   "Memory CD4 T"
```

We can find how to use scType here: https://github.com/IanevskiAleksandr/sc-type We adapt it so it returns:

- the predicted cell type
- a score of the prediction
- a matrix of the likelihood of each cell type for each cell. Cell types are rows and cells are columns.
- the list of the predicted cell type for each cell

Each classifier function has to be formated as follow to be used with the all the functions of the package, especially for advCGD.

```
   classifier = function(expr, clusters, target){
                c(
                    prediction="cell type",
                    odd=score,
                    typePredictions=my_matrix,
                    cellTypes=my_vector)
    }
```

```
scType_classifier = function(expr, clusters, target){
    expr = t(expr)
    library(HGNChelper)
```

```
source("https://raw.githubusercontent.com/IanevskiAleksandr/sc-type/master/R/sctype_s
core_.R")

source("https://raw.githubusercontent.com/IanevskiAleksandr/sc-type/master/R/gene_set
s_prepare.R")
    db_ =
"https://raw.githubusercontent.com/IanevskiAleksandr/sc-type/master/ScTypeDB_full.xls
x";
    tissue = "Immune system" # e.g. Immune
system,Pancreas,Liver,Eye,Kidney,Brain,Lung,Adrenal,Heart,Intestine,Muscle,Placenta,S
pleen,Stomach,Thymus
    # prepare gene sets
    gs_list <- gene_sets_prepare(db_, tissue)

    es.max = sctype_score(scRNAseqData = expr, scaled = T,
                          gs = gs_list$gs_positive, gs2 = gs_list$gs_negative)

    if (sum(clusters == target) == 0 ){
        return( c("UNDETERMINED",1))
    }
    cell_types <- apply(t(es.max[, clusters == target]), 1, function(x){
        names(x[x == max(x)])[1]
    })
    table_cell_type <- table(cell_types)
    str_class <- names(table_cell_type[order(table_cell_type, decreasing=T)][1])
    my_odd <- mean(es.max[str_class, clusters==target])/10
    resSCtype <- list(
        # Cell type prediction for the cluster
        prediction=str_class,
        # Score of the predicted cell type
        odd=my_odd,
        # Score for each cell type for each cell
        typePredictions=es.max,
        # Cell type for each cell
        cellTypes=cell_types)

    return(resSCtype)
}
```

We check if the classifier works properly by asking him to predict the “NK” cluster.

```
classifier_results <- scType_classifier(expr_df, clusters_df, "NK")
classifier_results$prediction

## [1] "Natural killer  cells"
```

The score of the prediction.

```
classifier_results$odd
```

```
## [1] 0.4461257
```

Likelihood of each cell type for each cell.

```
classifier_results$typePredictions[,clusters_df == "NK"][1:6,1:5]
```

```
##                     AAACCGTGTATGCG-1 AACGCCCTCGTACA-1 AAGATTACCTCAAG-1
## Pro-B cells              -0.05520064       0.03507093       0.16081207
## Pre-B cells              -0.59654036      -0.68228753      -0.44937559
## Naive B cells            -0.93855041      -0.04346109      -0.26012173
## Memory B cells           -1.00357418      -0.13690767      -0.34668843
## Plasma B cells           -0.60566221       0.28942711       0.07276648
## Naive CD8+ T cells        0.79524385       1.51477730       1.87037543
##                     AAGCAAGAGGTGTT-1 ACAAATTGTTGCGA-1
## Pro-B cells               -0.2828646        0.9118437
## Pre-B cells               -0.9487842        0.5254085
## Naive B cells             -1.2664716        1.7915419
## Memory B cells            -1.3210825        1.6398264
## Plasma B cells            -0.9335834        2.1244301
## Naive CD8+ T cells         0.9343186        0.2921489
```

Predicted cell type for each cell.

```
head(classifier_results$cellTypes)
```

```
##        AAACCGTGTATGCG-1        AACGCCCTCGTACA-1        AAGATTACCTCAAG-1
## "Natural killer  cells" "Natural killer  cells" "Natural killer  cells"
##        AAGCAAGAGGTGTT-1        ACAAATTGTTGCGA-1        ACAGGTACTGGTGT-1
##   "CD8+ NKT-like cells" "Natural killer  cells" "Natural killer  cells"
```

Let’s run a CGD attack

```
# Get significant genes
sign_genes <- getSignGenes(expr_df, clusters_df)
head(sign_genes$results)
```

```
##            gene         pval
## HLA.DRA HLA.DRA 1.004947e-70
## PRF1       PRF1 6.443834e-62
## NKG7       NKG7 1.070165e-79
## FCER1A   FCER1A 2.765930e-18
## TYROBP   TYROBP 8.127634e-85
```

```
## IL32       IL32 1.260937e-66
```

```r
# Run the attack
result_cgd <- advCGD(expr_df, clusters_df, "NK", classifier=scType_classifier ,
                     genes=sign_genes$results$gene[1:100] ,alpha=2, epsilon=2)
```

Modified genes

```r
result_cgd$modGenes
```

```
## [1] "NKG7" "GZMB"
```

Modified RNA expression matrix

```r
result_cgd$expr[1:5,1:5]
```

```
##                    AL627309.1  AP006222.2 RP11.206L10.2 RP11.206L10.9
## AAACATACAACCAC-1 -0.05812316 -0.03357571   -0.04166819   -0.03364562
## AAACATTGATCAGC-1 -0.05812316 -0.03357571   -0.04166819   -0.03364562
## AAACGCACTGGTAC-1 -0.05812316 -0.03357571   -0.04166819   -0.03364562
## AAATGTTGCCACAA-1 -0.05812316 -0.03357571   -0.04166819   -0.03364562
## AACACGTGCAGAGG-1 -0.05812316 -0.03357571   -0.04166819   -0.03364562
##                     LINC00115
## AAACATACAACCAC-1 -0.08223981
## AAACATTGATCAGC-1 -0.08223981
## AAACGCACTGGTAC-1 -0.08223981
## AAATGTTGCCACAA-1 -0.08223981
## AACACGTGCAGAGG-1 -0.08223981
```

Check the new classification of the result.

```r
new_classifier_results <- scType_classifier(result_cgd$expr, clusters_df, "NK")
new_classifier_results$prediction
```

```
## [1] "CD8+ NKT-like cells"
```

```r
sessionInfo()
```

```
## R version 4.3.0 (2023-04-21)
## Platform: x86_64-pc-linux-gnu (64-bit)
## Running under: Ubuntu 22.04.1 LTS
##
## Matrix products: default
## BLAS:   /usr/lib/x86_64-linux-gnu/blas/libblas.so.3.10.0
## LAPACK: /usr/lib/x86_64-linux-gnu/lapack/liblapack.so.3.10.0
##
```

```
## locale:
##  [1] LC_CTYPE=en_US.UTF-8       LC_NUMERIC=C
##  [3] LC_TIME=fr_FR.UTF-8        LC_COLLATE=en_US.UTF-8
##  [5] LC_MONETARY=fr_FR.UTF-8    LC_MESSAGES=en_US.UTF-8
##  [7] LC_PAPER=fr_FR.UTF-8       LC_NAME=C
##  [9] LC_ADDRESS=C               LC_TELEPHONE=C
## [11] LC_MEASUREMENT=fr_FR.UTF-8 LC_IDENTIFICATION=C
##
## time zone: Europe/Paris
## tzcode source: system (glibc)
##
## attached base packages:
## [1] stats4    stats     graphics  grDevices utils     datasets  methods
## [8] base
##
## other attached packages:
##  [1] HGNChelper_0.8.1            scran_1.29.0
##  [3] scater_1.29.0               scuttle_1.11.0
##  [5] CHETAH_1.17.0               ggplot2_3.4.2
##  [7] TENxPBMCData_1.19.0         HDF5Array_1.29.3
##  [9] rhdf5_2.45.0                DelayedArray_0.27.5
## [11] SparseArray_1.1.10          S4Arrays_1.1.4
## [13] Matrix_1.5-4.1              SingleCellExperiment_1.23.0
## [15] SummarizedExperiment_1.31.1 Biobase_2.61.0
## [17] GenomicRanges_1.53.1        GenomeInfoDb_1.37.2
## [19] IRanges_2.35.2              S4Vectors_0.39.1
## [21] BiocGenerics_0.47.0         MatrixGenerics_1.13.0
## [23] matrixStats_1.0.0           adverSCarial_1.3.6
##
## loaded via a namespace (and not attached):
##   [1] RColorBrewer_1.1-3            jsonlite_1.8.5
##   [3] magrittr_2.0.3                ggbeeswarm_0.7.2
##   [5] farver_2.1.1                  corrplot_0.92
##   [7] zlibbioc_1.47.0               vctrs_0.6.3
##   [9] memoise_2.0.1                 DelayedMatrixStats_1.23.0
##  [11] RCurl_1.98-1.12               htmltools_0.5.5
##  [13] AnnotationHub_3.9.1           curl_5.0.1
##  [15] BiocNeighbors_1.19.0          Rhdf5lib_1.23.0
##  [17] htmlwidgets_1.6.2             plyr_1.8.8
##  [19] plotly_4.10.2                 cachem_1.0.8
##  [21] igraph_1.5.0                  mime_0.12
##  [23] lifecycle_1.0.3               pkgconfig_2.0.3
##  [25] rsvd_1.0.5                    R6_2.5.1
##  [27] fastmap_1.1.1                 GenomeInfoDbData_1.2.10
##  [29] shiny_1.7.4                   digest_0.6.32
##  [31] colorspace_2.1-0              AnnotationDbi_1.63.1
##  [33] dqrng_0.3.0                   irlba_2.3.5.1
##  [35] ExperimentHub_2.8.0           RSQLite_2.3.1
```

```
##  [37] beachmat_2.17.8                 labeling_0.4.2
##  [39] filelock_1.0.2                  fansi_1.0.4
##  [41] httr_1.4.6                      compiler_4.3.0
##  [43] bit64_4.0.5                     withr_2.5.0
##  [45] BiocParallel_1.35.2             viridis_0.6.3
##  [47] DBI_1.1.3                       highr_0.10
##  [49] dendextend_1.17.1               rappdirs_0.3.3
##  [51] bluster_1.11.1                  tools_4.3.0
##  [53] vipor_0.4.5                     beeswarm_0.4.0
##  [55] interactiveDisplayBase_1.39.0 zip_2.3.0
##  [57] httpuv_1.6.11                   glue_1.6.2
##  [59] rhdf5filters_1.13.3             promises_1.2.0.1
##  [61] grid_4.3.0                      cluster_2.1.4
##  [63] reshape2_1.4.4                  generics_0.1.3
##  [65] gtable_0.3.3                    tidyr_1.3.0
##  [67] data.table_1.14.8               metapod_1.9.0
##  [69] BiocSingular_1.17.0             ScaledMatrix_1.9.1
##  [71] utf8_1.2.3                      XVector_0.41.1
##  [73] RcppAnnoy_0.0.20                ggrepel_0.9.3
##  [75] BiocVersion_3.18.0              pillar_1.9.0
##  [77] stringr_1.5.0                   limma_3.57.6
##  [79] later_1.3.1                     dplyr_1.1.2
##  [81] BiocFileCache_2.9.0             lattice_0.21-8
##  [83] bit_4.0.5                       tidyselect_1.2.0
##  [85] locfit_1.5-9.8                  Biostrings_2.69.1
##  [87] knitr_1.43                      gridExtra_2.3
##  [89] edgeR_3.43.7                    xfun_0.39
##  [91] statmod_1.5.0                   pheatmap_1.0.12
##  [93] stringi_1.7.12                  lazyeval_0.2.2
##  [95] yaml_2.3.7                      evaluate_0.21
##  [97] codetools_0.2-19                tibble_3.2.1
##  [99] BiocManager_1.30.21             cli_3.6.1
## [101] uwot_0.1.15                     xtable_1.8-4
## [103] munsell_0.5.0                   Rcpp_1.0.10
## [105] bioDist_1.73.0                  dbplyr_2.3.2
## [107] png_0.1-8                       parallel_4.3.0
## [109] ellipsis_0.3.2                  blob_1.2.4
## [111] sparseMatrixStats_1.13.0        bitops_1.0-7
## [113] viridisLite_0.4.2               scales_1.2.1
## [115] openxlsx_4.2.5.2                purrr_1.0.1
## [117] crayon_1.5.2                    rlang_1.1.1
## [119] cowplot_1.1.1                   KEGGREST_1.41.0
```

Supplementary Data B

Simulation of the single-cell dropout effect with an implementation of the custom modification.

```
fctDropout = function(x, c_mask){
   # Probability of dropout
   p_dropout <- 0.25
   exprs <- x[c_mask]
   drops <- rbinom(length(exprs), 1, 1 - p_dropout)
   return( exprs * drops )
}

# Use it to generate a single-gene attack
advSingleGene(exprs, clusters, target, classifier,
          advMethod="full_matrix_fct", advFct=fctDropout)
```

Supplementary Data C

single-gene attack mode algorithm.

```
Input:
        matrix: rna_expr
        list: cells_type
        string: cell_type_target
        function: classifier
        object: modification_type
        int: init_split_size
Output:
        list: results_attack_genes

Begin
   results_attack_genes <- empty list
   gene_groups <- groups of genes based on init_split_size

   For each group in list_gene_groups do
        results_attack_genes <- dichotSingleGenes(
                        results_attack_genes,
                        rna_expr,
                        cells_type,
                        cell_type_target,
                        function_classifier,
                        modification_type,
                        genes)

   Return list_results_attack_genes
End


Function dichotSingleGenes:
        Input:
                list: results_attack_genes
                matrix: rna_expr
                list: cells_type
                string: cell_type_target
                function: classifier
                object: modification_type
                list: genes
```

Output:
list: results_attack_genes

matrix: modified_rna_expr <- modified rna_expr matrix on genes with the modification modification_type

string: new_classification <- classification made by classifier on modified_rna_expr

```
If new_classification equals cell_type_target then
    return results_attack_genes

If length of genes is 1 then
    results_attack_genes[genes[0]] <- new_classification
    return results_attack_genes

list: genes_half1 <- one half of genes
list: genes_half2 <- other half of genes

results_attack_genes <- dichotSingleGenes(
    results_attack_genes,
    rna_expr,
    cells_type,
    cell_type_target,
    function_classifier,
    modification_type,
    genes_half1)

results_attack_genes <- dichotSingleGenes(
    results_attack_genes,
    rna_expr,
    cells_type,
    cell_type_target,
    function_classifier,
    modification_type,
    genes_half2)

return results_attack_genes
```

Supplementary Data D

max-change attack mode algorithm.

```
Input:
        matrix: rna_expr
        list: cells_type
        string: cell_type_target
        function: classifier
        object: modification_type

Output:
        list: results_attack_genes

Begin
        results_attack_genes <- empty list

        list: genes <- list of all gene names from matrix_rna_expr

        results_attack_genes <- dichotMaxChange(
                        results_attack_genes,
                        rna_expr,
                        cells_type,
                        cell_type_target,
                        classifier,
                        modification_type,
                        genes)

        return results_attack_genes
End

Function dichotMaxChange

        Input:
                list: results_attack_genes
                matrix: rna_expr
                list: cells_type
                string: cell_type_target
                function: classifier
                object: modification_type
                list: genes

        Output:
```

```
        list: results_attack_genes

    Begin
        matrix: modified_rna_expr <- rna_expr matrix with genes values modified with
the modification_type on the cell_type_target cells

        string: new_classification <- classification of the cell_type_target cells of
    modified_rna_expr with classifier

        If new_classification equals cell_type_target then
            list: concatenated_genes <- concatenate genes and
        results_attack_genes
            matrix: modified_rna_expr <- rna_expr matrix with
        concatenated_genes values modified with the modification_type on the
        cell_type_target cells
            string: new_classification <- classification of the cell_type_target cells
        of modified_rna_expr with classifier
            If new_classification equals cell_type_target then
                return concatenated_genes

        list: genes_half1 <- one half of genes
        list: genes_half2 <- other half of genes

        results_attack_genes <- dichotMaxChange(
            results_attack_genes,
            rna_expr,
            cells_type,
            cell_type_target,
            classifier,
            modification_type,
            genes)

        results_attack_genes <- dichotMaxChange(
            results_attack_genes,
            rna_expr,
            cells_type,
            cell_type_target,
            classifier,
            modification_type,
            genes)

        return results_attack_genes
```

Supplementary Data E

The function getSignGenes orders the genes by maximizing the significance of the gene to differentiate the clusters and ensures that they represent at most the variations across all possible pairs of clusters.

The getDistantCouples() function is used to generate all possible pairs of clusters and order them in a way that the following pairs are as distant as possible.

Example:

cell_types = c("B", "CD4 T", "NK", "CD8 T", "DC")

getDistantCouples(cell_types)

'B___CD4 T', 'NK___CD8 T', 'B___DC', 'CD4 T___NK',
'CD8 T___DC', 'B___NK', 'CD4 T___CD8 T', 'NK___DC',
'B___CD8 T', 'CD4 T___DC'

We loop through each pair from getDistantCouples(cell_types), selecting the most significantly differential gene between the two cell types in the pair (a gene not yet in the list), until all genes have been selected.

Parameters:

expr A matrix of gene expression data. Rows are cells and columns are genes.
clusters a character vector of the clusters to which the cells belong
method the statistical test to use. Either "wilcox" for the Wilcoxon rank sum test or "ttest" for the t-test. Default is "wilcox".
verbose logical, set to TRUE to activate verbose mode

```
getSignGenes <- function(expr, clusters, method="wilcox", verbose=FALSE){
      combinations <- getDistantCouples(unique(clusters))
      pvalList <- list()
      for(strComb in combinations){
            a_a <- unlist(strsplit(strComb, "___"))
            clust1 <- a_a[1]
            clust2 <- a_a[2]
            if (verbose) {message("Cluster ",clust1," vs ",clust2)}
            pvals <- apply(t(expr), 1, function(x){
                  c1 <- x[clusters == clust1]
                  c1 <- c1[!is.na(c1)]
                  c2 <- x[clusters == clust2]
                  c2 <- c2[!is.na(c2)]
                  if ( length(c1) == 0 || length(c2) == 0){
                        return(1)
```

```
                }
                # Handle constant values
                if (length(unique(c1))==1){
                        c1[1] = c1[1] + 0.01
                }
                # Handle constant values
                if (length(unique(c2))==1){
                        c2[1] = c2[1] + 0.01
                }
                if (method=="wilcox"){
                        return(wilcox.test(c1, c2)$p.value)
                }
                if (method=="ttest"){
                        return(t.test(c1, c2)$p.value)
                }
        })
        dfPvals <- data.frame(gene=colnames(expr), pval=unname(pvals))
        rownames(dfPvals) <- dfPvals$gene
        dfPvals <- dfPvals[order(dfPvals$pval),]
        pvalList[[strComb]] <- dfPvals
    }
    dfResults <- as.data.frame(matrix(nrow=0, ncol=2))
    colnames(dfResults) <- c("gene", "pval")
    for (i in 1:ncol(expr)){
        for(strCouple in names(pvalList)){
                strGene <- pvalList[[strCouple]][i,"gene"]
                if (!strGene %in% dfResults$gene){
                        dfResults <- rbind(dfResults,
                pvalList[[strCouple]][i,])
                }
        }
    }
    results <- list(results=dfResults, pvalList=pvalList)
    return(results)
}

getDistantCouples <- function(cell_types, verbose=FALSE){
    combinations <- combn(cell_types, 2, simplify = FALSE)
    strCombs <- c()
    for (i in combinations){
        strCombs <- c(strCombs, paste0(i[1], "___", i[2]))
    }
    twoPrevs <- c()
    selCombs <- c()
    intInd <- 1
    killLoop <- FALSE
    intKill <- 0
```

```
	while(length(selCombs)<length(combinations)){
		if (intKill == 0 && killLoop){
			selCombs <- c(selCombs, setdiff(strCombs, selCombs))
		}
		intKill <- intKill + 1
		killLoop <- TRUE
		c1 <- combinations[[intInd]][1]
		c2 <- combinations[[intInd]][2]
		myKey <- paste0(c1,"___",c2)

		if ( !c1 %in% twoPrevs && !c2 %in% twoPrevs && !myKey %in%
		selCombs){
			if (verbose){message(myKey)}
				intKill <- 0
				killLoop <- FALSE
				selCombs <- c(selCombs, myKey)
				twoPrevs <- c(c1, c2)
		}
		intInd <- intInd + 1
		if (intKill > length(combinations) +1){
			intKill <- 0
		}
		if (intInd > length(combinations)){
			intInd <- 1
		}
	}
	return(selCombs)
}
```

Supplementary Data F

Number of possible *single-gene* attacks and signature length of *max-change* attacks on different cell types, for several modifications, for scType on the *undivided* PBMC3K dataset.

	Single-gene / Max-change (signature length) attacks					
	decile+5	*perc1*	*perc99*	*aberrant_pos*	*aberrant_neg*	total
CD8+ NKT-like cells	2 / 0	5 / 9	23 / 31	158 / 158	33 / 33	248 / 1031
Classical monocytes	0 / 0	0 / 5	11 / 23	152 / 153	30 / 15	199 / 209
Myeloid dendritic cells	0 / 0	1 / 4	2 / 14	145 / 136	34 / 33	185 / 3031
Naive B cells	0 / 0	1 / 3	9 / 9	151 / 119	33 / 31	199 / 1443
Naive CD4+ T cells	1 / 5	1 / 4	40 / 54	149 / 142	29 / 29	267 / 6561
Natural killer cells	1 / 1	0 / 0	5 / 28	144 / 141	27 / 24	179 / 584
Non-classical monocytes	0 / 0	0 / 3	5 / 20	154 / 147	34 / 34	194 / 1993
Platelets	0 / 0	0 / 1	0 / 10	155 / 142	26 / 23	181 / 180
total	4 / 6	8 / 29	95 / 189	1208 / 1138	246 / 222	1652 / 15032

UMAP representations of the scType-annotated PBMC3k dataset, along with discriminating cell type markers, before and after single-gene or max-change attacks on the Naive B cell cluster.

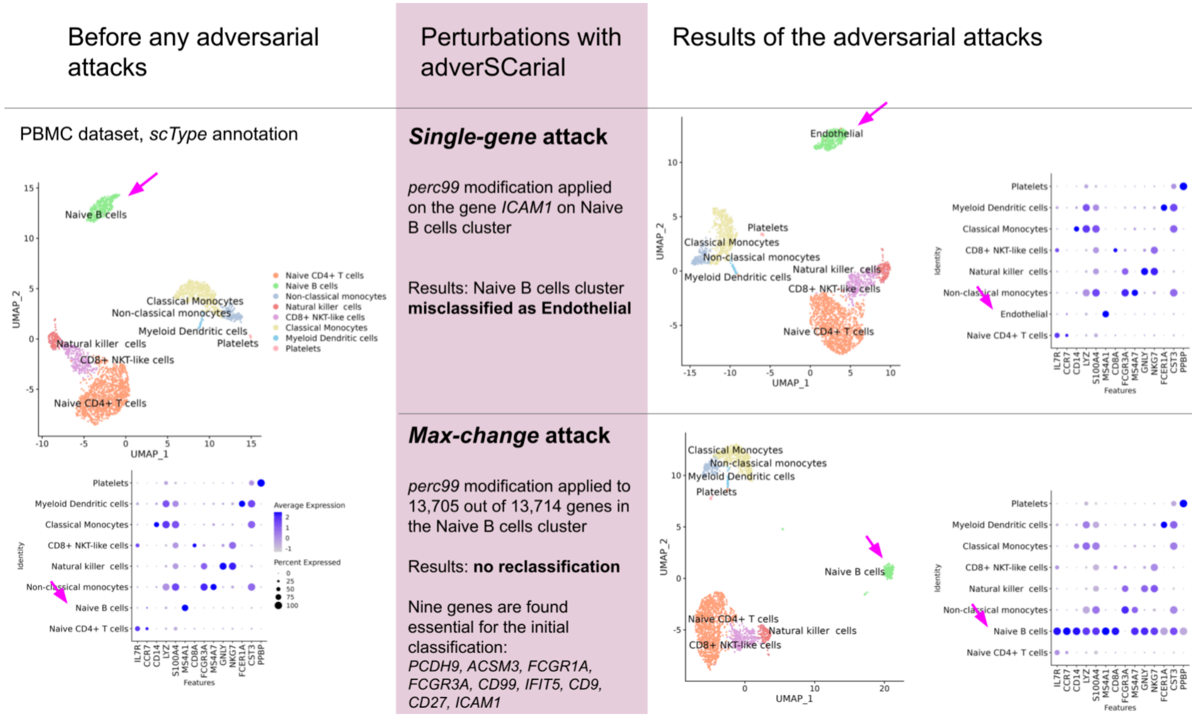

Supplementary Data G

Possible *single-gene* attacks with the *decile+5* modification on the *scType* classifier.

	original cell type	gene	new classification
1	B	*CD99*	Pro-B cells
2	DC	*FCER1A*	Mast cells
3	Naive CD4 T	*SSR4*	Plasma B cells
4	Naive CD4 T	*S100A4*	Memory CD4+ T cells
5	Naive CD4 T	*CD44*	Cancer cells
6	Naive CD4 T	*CXCR4*	Megakaryocyte
7	NK	*CD247*	CD8+ NKT-like cells

Supplementary Data H

Possible *single-gene* attacks with the *perc1* and *perc99* modification on the *scType*, *CHETAH*, *scAnnotatR*, *scRF* and *scMLP* classifier.

	original cell type	classifier	modification	gene	new classification
1	B	*scType*	*perc1*	*MS4A1*	Pre-B cells
2	B	*scType*	*perc99*	*FCER1A*	Mast cells
3	B	*scType*	*perc99*	*NSMCE1*	Pre-B cells
4	B	*scType*	*perc99*	*CD99*	Pro-B cells
5	B	*scType*	*perc99*	*KRT18*	Cancer cells
6	B	*scType*	*perc99*	*ICAM1*	Endothelial
7	B	*scType*	*perc99*	*CD9*	Pro-B cells
8	B	*scType*	*perc99*	*ACSM3*	Pre-B cells
9	B	*scType*	*perc99*	*CD27*	Memory B cells
10	B	*scType*	*perc99*	*TFRC*	Erythroid-like and erythroid precursor cells
11	CD14+ Mono	*scType*	*perc99*	*FCER1A*	Mast cells
12	CD14+ Mono	*scType*	*perc99*	*CD163*	Macrophages
13	CD14+ Mono	*scType*	*perc99*	*CDKN1C*	Non-classical monocytes
14	CD14+ Mono	*scType*	*perc99*	*KRT18*	Cancer cells
15	CD14+ Mono	*scType*	*perc99*	*FCGR3A*	Non-classical monocytes
16	CD14+ Mono	*scType*	*perc99*	*ICAM1*	Endothelial
17	CD14+ Mono	*scType*	*perc99*	*FCGR2B*	Macrophages
18	CD14+ Mono	*scType*	*perc99*	*TFRC*	Erythroid-like and erythroid precursor cells
19	CD8 T	*scType*	*perc1*	*NKG7*	Effector CD8+ T cells
20	CD8 T	*scType*	*perc1*	*CD8A*	Natural killer cells
21	CD8 T	*scType*	*perc1*	*CD3E*	Natural killer cells
22	CD8 T	*scType*	*perc1*	*CD3D*	Natural killer cells
23	CD8 T	*scType*	*perc99*	*FCER1A*	Mast cells
24	CD8 T	*scType*	*perc99*	*ISG20*	Effector CD8+ T cells
25	CD8 T	*scType*	*perc99*	*CD8B*	Effector CD8+ T cells
26	CD8 T	*scType*	*perc99*	*CLU*	Platelets
27	CD8 T	*scType*	*perc99*	*NT5E*	Progenitor cells
28	CD8 T	*scType*	*perc99*	*CD6*	Effector CD8+ T cells
29	CD8 T	*scType*	*perc99*	*KRT18*	Cancer cells
30	CD8 T	*scType*	*perc99*	*TNFRSF8*	Effector CD8+ T cells
31	CD8 T	*scType*	*perc99*	*RUVBL1*	Erythroid-like and erythroid precursor cells
32	CD8 T	*scType*	*perc99*	*CCR7*	Naive CD8+ T cells
33	CD8 T	*scType*	*perc99*	*SHISA5*	Effector CD8+ T cells
34	CD8 T	*scType*	*perc99*	*ICAM1*	Endothelial
35	CD8 T	*scType*	*perc99*	*FCGR2B*	Mast cells
36	CD8 T	*scType*	*perc99*	*CD36*	Erythroid-like and erythroid precursor cells
37	CD8 T	*scType*	*perc99*	*IL2RA*	Effector CD8+ T cells
38	CD8 T	*scType*	*perc99*	*ALDH1A1*	Progenitor cells
39	CD8 T	*scType*	*perc99*	*CCR6*	Effector CD8+ T cells
40	CD8 T	*scType*	*perc99*	*TFRC*	Erythroid-like and erythroid precursor cells
41	CD8 T	*scType*	*perc99*	*CCL3L3*	ISG expressing immune cells

42	CD8 T	*scType*	*perc99*	*ITGA4*	Erythroid-like and erythroid precursor cells
43	CD8 T	*scType*	*perc99*	*CD4*	CD4+ NKT-like cells
44	DC	*scType*	*perc1*	*CD1C*	Mast cells
45	DC	*scType*	*perc1*	*CLEC10A*	Mast cells
46	DC	*scType*	*perc99*	*FCGR2A*	Mast cells
47	DC	*scType*	*perc99*	*FCGR2B*	Mast cells
48	DC	*scType*	*perc99*	*IL2RA*	Mast cells
49	DC	*scType*	*perc99*	*CD33*	Mast cells
50	FCGR3A+ Mono	*scType*	*perc99*	*CD163*	Macrophages
51	FCGR3A+ Mono	*scType*	*perc99*	*KRT18*	Cancer cells
52	FCGR3A+ Mono	*scType*	*perc99*	*FCGR1A*	Macrophages
53	FCGR3A+ Mono	*scType*	*perc99*	*ICAM1*	Endothelial
54	FCGR3A+ Mono	*scType*	*perc99*	*FCGR2B*	Macrophages
55	Memory CD4 T	*scType*	*perc1*	*SELL*	γδ-T cells
56	Memory CD4 T	*scType*	*perc1*	*S100A4*	γδ-T cells
57	Memory CD4 T	*scType*	*perc1*	*ISG20*	Naive CD4+ T cells
58	Memory CD4 T	*scType*	*perc1*	*CD69*	Naive CD4+ T cells
59	Memory CD4 T	*scType*	*perc1*	*CD3E*	γδ-T cells
60	Memory CD4 T	*scType*	*perc1*	*CCR7*	γδ-T cells
61	Memory CD4 T	*scType*	*perc1*	*CD44*	Naive CD4+ T cells
62	Memory CD4 T	*scType*	*perc1*	*CD4*	γδ-T cells
63	Memory CD4 T	*scType*	*perc99*	*CD38*	Progenitor cells
64	Memory CD4 T	*scType*	*perc99*	*NKG7*	γδ-T cells
65	Memory CD4 T	*scType*	*perc99*	*GNLY*	Effector CD8+ T cells
66	Memory CD4 T	*scType*	*perc99*	*CD1C*	Myeloid Dendritic cells
67	Memory CD4 T	*scType*	*perc99*	*MNDA*	γδ-T cells
68	Memory CD4 T	*scType*	*perc99*	*FCER1A*	Mast cells
69	Memory CD4 T	*scType*	*perc99*	*CSF2RA*	γδ-T cells
70	Memory CD4 T	*scType*	*perc99*	*PF4*	Platelets
71	Memory CD4 T	*scType*	*perc99*	*CD8A*	Effector CD8+ T cells
72	Memory CD4 T	*scType*	*perc99*	*PPBP*	Platelets
73	Memory CD4 T	*scType*	*perc99*	*CD8B*	Effector CD8+ T cells
74	Memory CD4 T	*scType*	*perc99*	*ITGAX*	γδ-T cells
75	Memory CD4 T	*scType*	*perc99*	*CD163*	Macrophages
76	Memory CD4 T	*scType*	*perc99*	*SLAMF1*	HSC/MPP cells
77	Memory CD4 T	*scType*	*perc99*	*CLU*	Platelets
78	Memory CD4 T	*scType*	*perc99*	*GZMM*	γδ-T cells
79	Memory CD4 T	*scType*	*perc99*	*PAX5*	Plasma B cells
80	Memory CD4 T	*scType*	*perc99*	*NT5E*	Progenitor cells
81	Memory CD4 T	*scType*	*perc99*	*CLEC10A*	Myeloid Dendritic cells
82	Memory CD4 T	*scType*	*perc99*	*KRT18*	Cancer cells
83	Memory CD4 T	*scType*	*perc99*	*KLRB1*	CD4+ NKT-like cells
84	Memory CD4 T	*scType*	*perc99*	*CD19*	Pre-B cells
85	Memory CD4 T	*scType*	*perc99*	*IFIT2*	ISG expressing immune cells
86	Memory CD4 T	*scType*	*perc99*	*IFIT5*	ISG expressing immune cells
87	Memory CD4 T	*scType*	*perc99*	*RUVBL1*	Erythroid-like and erythroid precursor cells
88	Memory CD4 T	*scType*	*perc99*	*KLRD1*	CD4+ NKT-like cells
89	Memory CD4 T	*scType*	*perc99*	*RSAD2*	ISG expressing immune cells
90	Memory CD4 T	*scType*	*perc99*	*FCGR2A*	Mast cells
91	Memory CD4 T	*scType*	*perc99*	*FCGR3A*	γδ-T cells
92	Memory CD4 T	*scType*	*perc99*	*ICAM1*	Endothelial
93	Memory CD4 T	*scType*	*perc99*	*FCGR2B*	Mast cells
94	Memory CD4 T	*scType*	*perc99*	*ITGA6*	HSC/MPP cells
95	Memory CD4 T	*scType*	*perc99*	*ITGB1*	Progenitor cells
96	Memory CD4 T	*scType*	*perc99*	*STMN1*	Progenitor cells

97	Memory CD4 T	*scType*	*perc99*	*CD200*	Myeloid Dendritic cells
98	Memory CD4 T	*scType*	*perc99*	*CD36*	Erythroid-like and erythroid precursor cells
99	Memory CD4 T	*scType*	*perc99*	*ALDH1A1*	Progenitor cells
100	Memory CD4 T	*scType*	*perc99*	*CCR6*	γδ-T cells
101	Memory CD4 T	*scType*	*perc99*	*IGLL5*	Plasma B cells
102	Memory CD4 T	*scType*	*perc99*	*PRDX4*	Plasma B cells
103	Memory CD4 T	*scType*	*perc99*	*CD9*	Pro-B cells
104	Memory CD4 T	*scType*	*perc99*	*ACSM3*	Pre-B cells
105	Memory CD4 T	*scType*	*perc99*	*CD63*	Basophils
106	Memory CD4 T	*scType*	*perc99*	*TFRC*	Erythroid-like and erythroid precursor cells
107	Memory CD4 T	*scType*	*perc99*	*CCL4*	ISG expressing immune cells
108	Memory CD4 T	*scType*	*perc99*	*PTPRC*	γδ-T cells
109	Memory CD4 T	*scType*	*perc99*	*CCL3L3*	ISG expressing immune cells
110	Memory CD4 T	*scType*	*perc99*	*IL2RB*	CD4+ NKT-like cells
111	Memory CD4 T	*scType*	*perc99*	*ITGA4*	Erythroid-like and erythroid precursor cells
112	Memory CD4 T	*scType*	*perc99*	*OASL*	ISG expressing immune cells
113	Memory CD4 T	*scType*	*perc99*	*CD33*	Mast cells
114	Memory CD4 T	*scType*	*perc99*	*CD4*	Effector CD4+ T cells
115	Memory CD4 T	*scType*	*perc99*	*GNG11*	Platelets
116	Naive CD4 T	*scType*	*perc1*	*SELL*	γδ-T cells
117	Naive CD4 T	*scType*	*perc99*	*CD38*	Progenitor cells
118	Naive CD4 T	*scType*	*perc99*	*GNLY*	Naive CD8+ T cells
119	Naive CD4 T	*scType*	*perc99*	*CD79A*	Plasma B cells
120	Naive CD4 T	*scType*	*perc99*	*CD1C*	Myeloid Dendritic cells
121	Naive CD4 T	*scType*	*perc99*	*CD86*	Myeloid Dendritic cells
122	Naive CD4 T	*scType*	*perc99*	*S100A12*	Classical Monocytes
123	Naive CD4 T	*scType*	*perc99*	*CD40*	Myeloid Dendritic cells
124	Naive CD4 T	*scType*	*perc99*	*FCER1A*	Mast cells
125	Naive CD4 T	*scType*	*perc99*	*S100A4*	Memory CD4+ T cells
126	Naive CD4 T	*scType*	*perc99*	*ISG20*	Memory CD4+ T cells
127	Naive CD4 T	*scType*	*perc99*	*PF4*	Platelets
128	Naive CD4 T	*scType*	*perc99*	*CD8A*	Naive CD8+ T cells
129	Naive CD4 T	*scType*	*perc99*	*PPBP*	Platelets
130	Naive CD4 T	*scType*	*perc99*	*CD8B*	Naive CD8+ T cells
131	Naive CD4 T	*scType*	*perc99*	*ITGAM*	Eosinophils
132	Naive CD4 T	*scType*	*perc99*	*ITGAX*	Myeloid Dendritic cells
133	Naive CD4 T	*scType*	*perc99*	*CD163*	Macrophages
134	Naive CD4 T	*scType*	*perc99*	*SLAMF1*	HSC/MPP cells
135	Naive CD4 T	*scType*	*perc99*	*SEC11C*	Plasma B cells
136	Naive CD4 T	*scType*	*perc99*	*CD83*	Myeloid Dendritic cells
137	Naive CD4 T	*scType*	*perc99*	*CLU*	Platelets
138	Naive CD4 T	*scType*	*perc99*	*PAX5*	Plasma B cells
139	Naive CD4 T	*scType*	*perc99*	*NT5E*	Progenitor cells
140	Naive CD4 T	*scType*	*perc99*	*CLEC10A*	Myeloid Dendritic cells
141	Naive CD4 T	*scType*	*perc99*	*FAS*	Memory CD4+ T cells
142	Naive CD4 T	*scType*	*perc99*	*KRT18*	Cancer cells
143	Naive CD4 T	*scType*	*perc99*	*CD19*	Pre-B cells
144	Naive CD4 T	*scType*	*perc99*	*IFIT2*	ISG expressing immune cells
145	Naive CD4 T	*scType*	*perc99*	*CD69*	HSC/MPP cells
146	Naive CD4 T	*scType*	*perc99*	*IFIT1*	ISG expressing immune cells
147	Naive CD4 T	*scType*	*perc99*	*IFIT5*	ISG expressing immune cells
148	Naive CD4 T	*scType*	*perc99*	*FCGR1A*	Macrophages
149	Naive CD4 T	*scType*	*perc99*	*RUVBL1*	Erythroid-like and erythroid precursor cells

150 Naive CD4 T	*scType*	*perc99*	*CD44*	Cancer cells
151 Naive CD4 T	*scType*	*perc99*	*KLRD1*	CD4+ NKT-like cells
152 Naive CD4 T	*scType*	*perc99*	*RSAD2*	ISG expressing immune cells
153 Naive CD4 T	*scType*	*perc99*	*FCGR2A*	Mast cells
154 Naive CD4 T	*scType*	*perc99*	*ICAM1*	Endothelial
155 Naive CD4 T	*scType*	*perc99*	*FCGR2B*	Mast cells
156 Naive CD4 T	*scType*	*perc99*	*ITGA6*	HSC/MPP cells
157 Naive CD4 T	*scType*	*perc99*	*ITGB1*	Progenitor cells
158 Naive CD4 T	*scType*	*perc99*	*STMN1*	Progenitor cells
159 Naive CD4 T	*scType*	*perc99*	*CD200*	Myeloid Dendritic cells
160 Naive CD4 T	*scType*	*perc99*	*CD36*	Erythroid-like and erythroid precursor cells
161 Naive CD4 T	*scType*	*perc99*	*IL2RA*	Mast cells
162 Naive CD4 T	*scType*	*perc99*	*ALDH1A1*	Progenitor cells
163 Naive CD4 T	*scType*	*perc99*	*IGLL5*	Plasma B cells
164 Naive CD4 T	*scType*	*perc99*	*PRDX4*	Plasma B cells
165 Naive CD4 T	*scType*	*perc99*	*CD9*	Pro-B cells
166 Naive CD4 T	*scType*	*perc99*	*ACSM3*	Pre-B cells
167 Naive CD4 T	*scType*	*perc99*	*CD63*	Basophils
168 Naive CD4 T	*scType*	*perc99*	*VPREB3*	Plasma B cells
169 Naive CD4 T	*scType*	*perc99*	*TFRC*	Erythroid-like and erythroid precursor cells
170 Naive CD4 T	*scType*	*perc99*	*CD14*	HSC/MPP cells
171 Naive CD4 T	*scType*	*perc99*	*TCL1A*	Plasma B cells
172 Naive CD4 T	*scType*	*perc99*	*FKBP11*	Plasma B cells
173 Naive CD4 T	*scType*	*perc99*	*CCL3*	ISG expressing immune cells
174 Naive CD4 T	*scType*	*perc99*	*CCL4*	ISG expressing immune cells
175 Naive CD4 T	*scType*	*perc99*	*CCL3L3*	ISG expressing immune cells
176 Naive CD4 T	*scType*	*perc99*	*CXCR4*	Megakaryocyte
177 Naive CD4 T	*scType*	*perc99*	*ITGA4*	Erythroid-like and erythroid precursor cells
178 Naive CD4 T	*scType*	*perc99*	*OASL*	ISG expressing immune cells
179 Naive CD4 T	*scType*	*perc99*	*CD33*	Mast cells
180 Naive CD4 T	*scType*	*perc99*	*GNG11*	Platelets
181 NK	*scType*	*perc99*	*CD8A*	CD8+ NKT-like cells
182 NK	*scType*	*perc99*	*CD3E*	CD8+ NKT-like cells
183 NK	*scType*	*perc99*	*CD3D*	CD8+ NKT-like cells
184 NK	*scType*	*perc99*	*CD3G*	CD8+ NKT-like cells
185 NK	*scType*	*perc99*	*CD4*	CD4+ NKT-like cells
186 Memory CD4 T	*CHETAH*	*perc99*	*NKG7*	CD8 T
187 Memory CD4 T	*CHETAH*	*perc99*	*CCL5*	CD8 T
188 Memory CD4 T	*scAnnotatR*	*perc1*	*S100A4*	Naive_CD4_T
189 Memory CD4 T	*scAnnotatR*	*perc1*	*LTB*	Naive_CD4_T
190 Memory CD4 T	*scAnnotatR*	*perc99*	*AIF1*	Naive_CD4_T
191 Memory CD4 T	*scAnnotatR*	*perc99*	*LGALS2*	Naive_CD4_T
192 Naive CD4 T	*scAnnotatR*	*perc1*	*RPS27*	Memory_CD4_T
193 Naive CD4 T	*scAnnotatR*	*perc99*	*HLA-DPB1*	Memory_CD4_T

Supplementary Data I

Time to compute a modification on a single gene on a cluster according the computer specification.

	dataset	cell_type	run_time_seconds_8Go	run_time_seconds_64Go
1	AXILLA10k	T cell	6.9075842	0.7506056
2	AXILLA10k	blood vessel smooth muscle cell	7.4472220	0.7516716
3	AXILLA10k	macrophage	4.3051889	0.7706778
4	AXILLA10k	fibroblast	19.4733021	0.7479694
5	AXILLA10k	blood vessel endothelial cell	11.1207747	0.7506957
6	AXILLA10k	malignant cell	16.5087471	0.7430904
7	LIVER10k	mature NK T cell	8.0492675	0.7839687
8	LIVER10k	monocyte	2.1946607	0.7520194
9	LIVER10k	macrophage	8.5248697	0.7617826
10	LIVER10k	blood vessel smooth muscle cell	1.9420846	2.3856821
11	LIVER10k	endothelial cell	1.7951071	0.7212138
12	LIVER10k	malignant cell	2.2242737	0.7918675
13	KIDNEY10k	kidney loop of Henle epithelial cell	5.9940536	1.8280592
14	KIDNEY10k	mesenchymal cell	12.9182546	4.6424403
15	KIDNEY10k	epithelial cell of proximal tubule	2.7780950	2.1197126
16	KIDNEY10k	podocyte	13.1573253	0.9578941
17	KIDNEY10k	kidney cell	8.3272038	0.9710867
18	KIDNEY10k	kidney epithelial cell	38.3660998	0.9931834
19	PBMC3k	Platelet	2.1972752	0.4697165
20	PBMC3k	DC	5.6388071	0.4775803
21	PBMC3k	FCGR3A+ Mono	1.1574850	0.4660211
22	PBMC3k	Naive CD4 T	0.8134842	0.4634564
23	PBMC3k	CD8 T	19.0655210	0.4895785
24	PBMC3k	NK	0.5502934	0.4705262
25	PBMC3k	CD14+ Mono	4.3796666	0.4780073
26	PBMC3k	B	31.8662832	0.5913293
27	PBMC3k	Memory CD4 T	8.2440343	3.8227537

Supplementary Data J

Time to compute a classification on a cluster according the classifier and the computer specification.

	classifier	dataset	cell_type	run_time_seconds_8Go	run_time_seconds_64Go
1	scType	PBMC3k	Memory CD4 T	10.130424	7.128746
2	scType	PBMC3k	B	49.430787	4.456954
3	scType	PBMC3k	CD14+ Mono	100.734941	4.294293
4	scType	PBMC3k	NK	67.320230	4.306873
5	scType	PBMC3k	CD8 T	3.634805	4.239968
6	scType	PBMC3k	Naive CD4 T	3.875137	3.820143
7	scType	PBMC3k	FCGR3A+ Mono	4.435320	3.905598
8	scType	PBMC3k	DC	3.627005	3.734745
9	scType	PBMC3k	Platelet	33.754122	3.896813
10	scType	KIDNEY10k	kidney epithelial cell	563.734092	87.003175
11	scType	KIDNEY10k	kidney cell	654.527555	92.547793
12	scType	KIDNEY10k	podocyte	592.427072	82.662987
13	scType	KIDNEY10k	epithelial cell of proximal tubule	544.445863	89.045865
14	scType	KIDNEY10k	mesenchymal cell	628.713053	94.221057
15	scType	KIDNEY10k	kidney loop of Henle epithelial cell	571.844440	86.695149
16	scType	LIVER10k	malignant cell	618.313565	72.131395
17	scType	LIVER10k	endothelial cell	524.869064	76.658090
18	scType	LIVER10k	blood vessel smooth muscle cell	380.206414	69.986727
19	scType	LIVER10k	macrophage	638.593372	70.517118
20	scType	LIVER10k	monocyte	465.349015	64.989053
21	scType	LIVER10k	mature NK T cell	556.617122	68.292509
22	scType	AXILLA10k	malignant cell	796.506907	73.721148
23	scType	AXILLA10k	blood vessel endothelial cell	1902.897680	67.128047
24	scType	AXILLA10k	fibroblast	630.601105	73.251773
25	scType	AXILLA10k	macrophage	662.199738	74.382884
26	scType	AXILLA10k	blood vessel smooth muscle cell	537.098116	72.473697
27	scType	AXILLA10k	T cell	1973.468118	71.777240
28	CHETAH	PBMC3k	Memory CD4 T	55.732468	22.419087
29	CHETAH	PBMC3k	B	61.379519	16.722304
30	CHETAH	PBMC3k	CD14+ Mono	18.412206	15.432330
31	CHETAH	PBMC3k	NK	27.713616	16.816110
32	CHETAH	PBMC3k	CD8 T	19.943627	16.438788
33	CHETAH	PBMC3k	Naive CD4 T	19.432557	16.821759
34	CHETAH	PBMC3k	FCGR3A+ Mono	36.071446	17.036295
35	CHETAH	PBMC3k	DC	28.369812	16.141948
36	CHETAH	PBMC3k	Platelet	43.990552	16.626541
37	CHETAH	KIDNEY10k	kidney epithelial cell	611.805054	42.572012
38	CHETAH	KIDNEY10k	kidney cell	388.662649	38.798212
39	CHETAH	KIDNEY10k	podocyte	314.729123	38.409959

	classifier	dataset	cell_type	run_time_seconds_8Go	run_time_seconds_64Go
40	CHETAH	KIDNEY10k	epithelial cell of proximal tubule	668.638172	38.649919
41	CHETAH	KIDNEY10k	mesenchymal cell	378.307584	38.611716
42	CHETAH	KIDNEY10k	kidney loop of Henle epithelial cell	272.838702	39.191972
43	CHETAH	LIVER10k	malignant cell	378.015843	36.801069
44	CHETAH	LIVER10k	endothelial cell	371.274626	32.421805
45	CHETAH	LIVER10k	blood vessel smooth muscle cell	403.667134	34.095156
46	CHETAH	LIVER10k	macrophage	309.960938	32.961097
47	CHETAH	LIVER10k	monocyte	378.567890	34.025957
48	CHETAH	LIVER10k	mature NK T cell	285.708393	34.357596
49	CHETAH	AXILLA10k	malignant cell	346.649204	37.600334
50	CHETAH	AXILLA10k	blood vessel endothelial cell	379.117607	34.558070
51	CHETAH	AXILLA10k	fibroblast	366.497538	32.500748
52	CHETAH	AXILLA10k	macrophage	312.944624	32.788214
53	CHETAH	AXILLA10k	blood vessel smooth muscle cell	161.859213	35.019002
54	CHETAH	AXILLA10k	T cell	234.534557	33.537247
55	scAnnotatR	PBMC3k	Memory CD4 T	3.087349	8.361531
56	scAnnotatR	PBMC3k	B	3.158466	2.731389
57	scAnnotatR	PBMC3k	CD14+ Mono	3.864009	2.484763
58	scAnnotatR	PBMC3k	NK	3.086731	2.713589
59	scAnnotatR	PBMC3k	CD8 T	3.145735	2.557976
60	scAnnotatR	PBMC3k	Naive CD4 T	3.111560	2.723223
61	scAnnotatR	PBMC3k	FCGR3A+ Mono	3.149391	2.981153
62	scAnnotatR	PBMC3k	DC	3.794687	2.734866
63	scAnnotatR	PBMC3k	Platelet	13.421275	2.502826
64	scAnnotatR	KIDNEY10k	kidney epithelial cell	714.402565	35.793123
65	scAnnotatR	KIDNEY10k	kidney cell	731.889410	36.968775
66	scAnnotatR	KIDNEY10k	podocyte	1258.492596	39.844038
67	scAnnotatR	KIDNEY10k	epithelial cell of proximal tubule	1125.695970	37.895170
68	scAnnotatR	KIDNEY10k	mesenchymal cell	1029.444901	33.174247
69	scAnnotatR	KIDNEY10k	kidney loop of Henle epithelial cell	1103.232669	36.692938
70	scAnnotatR	LIVER10k	malignant cell	616.505394	21.203533
71	scAnnotatR	LIVER10k	endothelial cell	431.960562	20.269569
72	scAnnotatR	LIVER10k	blood vessel smooth muscle cell	341.866044	24.654787
73	scAnnotatR	LIVER10k	macrophage	618.532580	20.444594
74	scAnnotatR	LIVER10k	monocyte	332.876665	17.896245
75	scAnnotatR	LIVER10k	mature NK T cell	580.831894	20.057257
76	scAnnotatR	AXILLA10k	malignant cell	874.980423	25.843534
77	scAnnotatR	AXILLA10k	blood vessel endothelial cell	857.493401	29.034171
78	scAnnotatR	AXILLA10k	fibroblast	959.829151	35.791260
79	scAnnotatR	AXILLA10k	macrophage	891.484807	32.673366
80	scAnnotatR	AXILLA10k	blood vessel smooth muscle cell	867.754949	31.104955
81	scAnnotatR	AXILLA10k	T cell	498.839635	31.529672
82	RF	PBMC3k	Memory CD4 T	1.551171	1.381554
83	RF	PBMC3k	B	5.689641	1.421097
84	RF	PBMC3k	CD14+ Mono	15.059407	1.292994
85	RF	PBMC3k	NK	1.526062	1.348964
86	RF	PBMC3k	CD8 T	12.080061	1.543160
87	RF	PBMC3k	Naive CD4 T	14.836613	1.363703

	classifier	dataset	cell_type	run_time_seconds_8Go	run_time_seconds_64Go
88	RF	PBMC3k	FCGR3A+ Mono	3.800665	1.834652
89	RF	PBMC3k	DC	1.516302	1.277641
90	RF	PBMC3k	Platelet	1.905782	1.829473
91	RF	KIDNEY10k	kidney epithelial cell	278.402273	19.757225
92	RF	KIDNEY10k	kidney cell	318.574690	22.541270
93	RF	KIDNEY10k	podocyte	231.642206	26.199915
94	RF	KIDNEY10k	epithelial cell of proximal tubule	695.203075	22.559423
95	RF	KIDNEY10k	mesenchymal cell	375.653268	25.757101
96	RF	KIDNEY10k	kidney loop of Henle epithelial cell	409.810131	23.023056
97	RF	LIVER10k	malignant cell	272.824515	15.720702
98	RF	LIVER10k	endothelial cell	281.444054	18.981113
99	RF	LIVER10k	blood vessel smooth muscle cell	225.471933	14.560827
100	RF	LIVER10k	macrophage	293.076638	12.987240
101	RF	LIVER10k	monocyte	258.291583	15.728158
102	RF	LIVER10k	mature NK T cell	303.796989	14.330949
103	RF	AXILLA10k	malignant cell	360.035328	18.918857
104	RF	AXILLA10k	blood vessel endothelial cell	264.807976	17.875348
105	RF	AXILLA10k	fibroblast	295.817076	16.913344
106	RF	AXILLA10k	macrophage	414.883500	18.017020
107	RF	AXILLA10k	blood vessel smooth muscle cell	251.071668	16.354191
108	RF	AXILLA10k	T cell	376.933928	18.859460
109	MLP	PBMC3k	Memory CD4 T	5.114492	11.612850
110	MLP	PBMC3k	B	5.834571	1.203523
111	MLP	PBMC3k	CD14+ Mono	4.591908	1.231724
112	MLP	PBMC3k	NK	5.429606	1.433955
113	MLP	PBMC3k	CD8 T	16.435067	1.232599
114	MLP	PBMC3k	Naive CD4 T	1.883815	1.468306
115	MLP	PBMC3k	FCGR3A+ Mono	2.876599	1.221308
116	MLP	PBMC3k	DC	1.729083	1.232549
117	MLP	PBMC3k	Platelet	4.441856	1.442447
118	MLP	KIDNEY10k	kidney epithelial cell	291.885531	23.219886
119	MLP	KIDNEY10k	kidney cell	174.669202	22.110513
120	MLP	KIDNEY10k	podocyte	170.590117	18.012353
121	MLP	KIDNEY10k	epithelial cell of proximal tubule	297.945899	19.866998
122	MLP	KIDNEY10k	mesenchymal cell	303.774585	22.180278
123	MLP	KIDNEY10k	kidney loop of Henle epithelial cell	237.773430	20.904613
124	MLP	LIVER10k	malignant cell	115.055140	14.835009
125	MLP	LIVER10k	endothelial cell	179.005166	15.431049
126	MLP	LIVER10k	blood vessel smooth muscle cell	318.098068	13.540138
127	MLP	LIVER10k	macrophage	175.024044	14.468637
128	MLP	LIVER10k	monocyte	209.386986	12.953182
129	MLP	LIVER10k	mature NK T cell	106.518395	12.921965
130	MLP	AXILLA10k	malignant cell	213.884771	23.390145
131	MLP	AXILLA10k	blood vessel endothelial cell	316.758781	13.699355
132	MLP	AXILLA10k	fibroblast	192.994326	18.433421
133	MLP	AXILLA10k	macrophage	156.780317	16.843600
134	MLP	AXILLA10k	blood vessel smooth muscle cell	194.882024	17.166422
135	MLP	AXILLA10k	T cell	33.016447	17.996161

Supplementary Data K

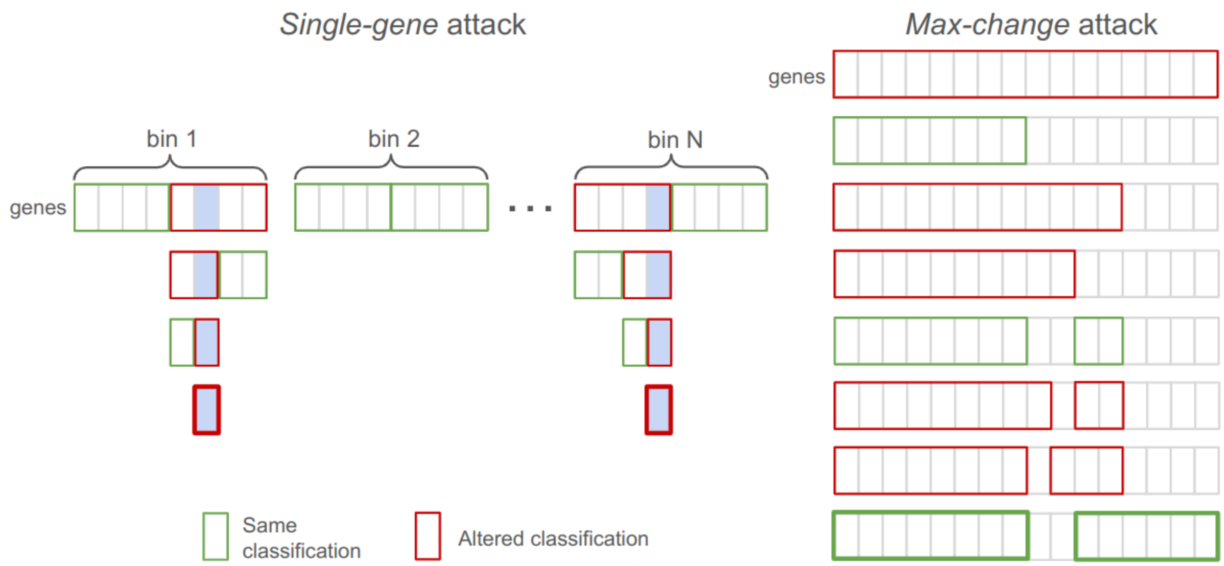

Supplementary Data L

Overview analysis with *adverSCarial*.

Load data

```
library(adverSCarial)
library(LoomExperiment)
library(DelayedArray)
```

Load loom file

```
pbmcPath <- system.file("extdata", "pbmc_short.loom", package="adverSCarial")
lfile <- import(pbmcPath, type="SingleCellLoomExperiment")
```

```
matPbmc <- counts(lfile)
```

```
matPbmc[1:5,1:5]
```

```
## <5 x 5> DelayedMatrix object of type "integer":
##      NEK11 IL5RA OR4C5 KHDRBS3 OPA3
## [1,]     0     0     0       0    0
## [2,]     0     0     0       0    0
## [3,]     0     0     0       0    0
## [4,]     0     0     0       0    0
## [5,]     0     0     0       0    0
```

Load cell type annotations

```
cellTypes <- rowData(lfile)$cell_type
```

```
head(cellTypes)
```

```
## [1] "Naive CD4 T"  "Naive CD4 T"  "FCGR3A+ Mono" "Naive CD4 T"  "B"
## [6] "Naive CD4 T"
```

Run vulnerability analysis with `singleGeneOverview` and `maxChangeOverview`

The `singleGeneOverview` and `maxChangeOverview` functions are designed to provide insight into of the min and max change adversarial attacks on each cell type, on various gene modifications.

Which attack to choose?

Before generating an attack it is judicious to choose the cell type to attack, and the modification susceptible to lead to a successful attack. Both functions run attack approximations, faster than the original, by studying splices of 100 genes.

Which classifier is more vulnerable to adversarial attacks?

Sometimes we want to compare two classifiers and see which one is more vulnerable to adversarial attacks.

Which modifications to compare

Here we define the modifications to analyse, the predefined `perc1`, and a custom function returning high outliers called `modifOutlier`.

```
modifOutlier <- function(x, y){
    return (max(x)*1000)
}

modifications <- list(c("perc1"), c("full_row_fct", modifOutlier))
```

We run the `singleGeneOverview`, this gives us a general idea of which cell types are more vulnerable to single gene attacks.

```
min_change_overview <- singleGeneOverview(matPbmc, cellTypes, MClassifier,
    modifications= modifications, maxSplitSize = 20, firstDichot = 5)

min_change_overview
```

```
## DataFrame with 10 rows and 2 columns
##                   perc1
##               <numeric>
## Naive CD4 T           1
```

```
## FCGR3A+ Mono         1
## B                    1
## Memory CD4 T         1
## CD14+ Mono           1
## CD8 T                1
## UNDETERMINED         0
## NK                   1
## Platelet             1
## DC                   1
##              full_row_fct_function..x..y.........return.max.x....1000...
##                                                            <numeric>
## Naive CD4 T                                                        3
## FCGR3A+ Mono                                                       2
## B                                                                  7
## Memory CD4 T                                                       8
## CD14+ Mono                                                         6
## CD8 T                                                              4
## UNDETERMINED                                                       8
## NK                                                                 5
## Platelet                                                           0
## DC                                                                 1
```

And the `maxChangeOverview`, giving us a general idea of which cell types are more vulnerable to max change attacks.

```
max_change_overview <- maxChangeOverview(matPbmc, cellTypes, MClassifier,
    modifications= modifications, maxSplitSize = 20)
```

```
max_change_overview
```

```
## DataFrame with 10 rows and 2 columns
##                    perc1
##                <numeric>
## Naive CD4 T            1
## FCGR3A+ Mono           1
## B                      1
## Memory CD4 T           1
## CD14+ Mono             1
## CD8 T                  1
## UNDETERMINED           1
## NK                     1
## Platelet               1
## DC                     1
##              full_row_fct_function..x..y.........return.max.x....1000...
##                                                            <numeric>
## Naive CD4 T                                                      163
## FCGR3A+ Mono                                                     176
```

```
## B                                                                     112
## Memory CD4 T                                                           99
## CD14+ Mono                                                            125
## CD8 T                                                                 151
## UNDETERMINED                                                           86
## NK                                                                    138
## Platelet                                                              200
## DC                                                                    188
```

```
sessionInfo()
```

```
## R version 4.4.1 (2024-06-14)
## Platform: x86_64-pc-linux-gnu
## Running under: Ubuntu 22.04.4 LTS
## 
## Matrix products: default
## BLAS:   /home/biocbuild/bbs-3.20-bioc/R/lib/libRblas.so 
## LAPACK: /usr/lib/x86_64-linux-gnu/lapack/liblapack.so.3.10.0
## 
## locale:
##  [1] LC_CTYPE=en_US.UTF-8       LC_NUMERIC=C              
##  [3] LC_TIME=en_GB              LC_COLLATE=C              
##  [5] LC_MONETARY=en_US.UTF-8    LC_MESSAGES=en_US.UTF-8   
##  [7] LC_PAPER=en_US.UTF-8       LC_NAME=C                 
##  [9] LC_ADDRESS=C               LC_TELEPHONE=C            
## [11] LC_MEASUREMENT=en_US.UTF-8 LC_IDENTIFICATION=C       
## 
## time zone: America/New_York
## tzcode source: system (glibc)
## 
## attached base packages:
## [1] stats4    stats     graphics  grDevices utils     datasets  methods  
## [8] base     
## 
## other attached packages:
##  [1] DelayedArray_0.31.7         SparseArray_1.5.18         
##  [3] S4Arrays_1.5.4              abind_1.4-5                
##  [5] Matrix_1.7-0                LoomExperiment_1.23.0      
##  [7] BiocIO_1.15.0               rhdf5_2.49.0               
##  [9] SingleCellExperiment_1.27.2 SummarizedExperiment_1.35.1
## [11] Biobase_2.65.0              GenomicRanges_1.57.1       
## [13] GenomeInfoDb_1.41.1         IRanges_2.39.1             
## [15] MatrixGenerics_1.17.0       matrixStats_1.3.0          
## [17] S4Vectors_0.43.1            BiocGenerics_0.51.0        
## [19] adverSCarial_1.3.9         
## 
## loaded via a namespace (and not attached):
##  [1] jsonlite_1.8.8          compiler_4.4.1          crayon_1.5.3
```

```
##  [4] stringr_1.5.1          rhdf5filters_1.17.0     yaml_2.3.9
##  [7] lattice_0.22-6         R6_2.5.1                XVector_0.45.0
## [10] commonmark_1.9.1       knitr_1.48              GenomeInfoDbData_1.2.12
## [13] rlang_1.1.4            stringi_1.8.4           HDF5Array_1.33.3
## [16] xfun_0.45              cli_3.6.3               magrittr_2.0.3
## [19] Rhdf5lib_1.27.0        zlibbioc_1.51.1         grid_4.4.1
## [22] markdown_1.13          lifecycle_1.0.4         glue_1.7.0
## [25] evaluate_0.24.0        httr_1.4.7              tools_4.4.1
## [28] UCSC.utils_1.1.0
```

Supplementary Data M

Classifier	Cell type	Attack mode	Modification	Genes of interest	Differential statistics
CHETAH	CD14+ mono	*Max-change*	*perc1*	EEF1A1, FOS, HLA-DPB1, **LAPTM5**, LGALS1, ST1, LYZ, OAZ1, PFDN5, RPL13A, RPL21, **RPL29**, **RPS11**, RPS15A, RPS23, RPS27A, RPS4X, PS4Y1, RPS5, S100A10, S100A4, S100A8, TPT1, TYROBP, UBA52, **RN7SK**	**see below**
scRF	CD8 T	*Max-change*	*perc99*	**BI3**, ACTN1, AIF1, AP1S2, ARPC3, CAPG, CD3G, CST3, CTSS, **CYTH4**, **DENND6B**, **ENO1**, FTH1, **GIMAP4**, GRN, HLA-DMA, **KLF2**, LGALS2, **LGALS3**, **LINC00938**, PGD, **PSMA7**, **PTP4A2**, **AD23A**, **RP11-220I1**, SEMA4A, SERPINA1, **SFR1**, **LC30A7**, **SND1**, **SPAG7**, SPINT2, TIMP1, **TTC38**, **UBALD2**, **WDR83OS**	**see below**
scAnnotatR	Platelet	*Single-gene*	*Negative-aberrant*	**IF1**, **CST3**, **HLA-DPB1**, HLA-DRB1, **LGALS1**, **LYZ**, MALAT1, S100A4, **S100A8**, **S100A9**, **TYMP**, **TYROBP**	**see below**

Table 1. Examples of discriminating genes uncovered by adversarial attacks. Genes that were not significant with standard differential statistics are highlighted in bold (adjusted p-value < 0.05; bilateral Student's test on targeted cells vs other cells followed by Benjamini-Hochberg correction for multi-testing).

CHETAH

perc1 modification *max-change* signature of 26 genes on the CD14+ mono cell type with *CHETAH*, among which 4 are not significantly different.

	p-value	FDR (Benjamini-Hochberg)
EEF1A1	6.23869071900704E-22	4.3651737000236E-20
FOS	1.10437995274325E-06	1.64267534402614E-05
HLA-DPB1	1.68757635395306E-17	8.97031865043111E-16
LAPTM5	0.144645710209995	0.309019774448152
LGALS1	3.58015415262522E-193	8.18303900818372E-190
LST1	1.33950706213731E-145	1.66999998637738E-142
LYZ	0	0
OAZ1	9.48783521436832E-109	6.50580860649236E-106
PFDN5	2.21696507219841E-35	3.13437721650814E-33
RPL13A	9.03075794019382E-40	1.4921423420701E-37
RPL21	3.96574674172095E-50	9.06437513599351E-48
RPL29	0.84719871294591	0.921078416786127
RPS11	0.192376037191384	0.360613036364495
RPS15A	3.7954847090538E-27	3.61467203471971E-25
RPS23	1.66684395469092E-26	1.51384754931333E-24
RPS27A	1.88661161562398E-53	4.88169654654099E-51
RPS4X	5.6878943093572E-20	3.66214941589317E-18

RPS4Y1	0.010526476371922	0.045084352581055
RPS5	1.95568705686762E-19	1.20270369048801E-17
S100A10	4.99554883294326E-23	3.76422838983428E-21
S100A4	2.91777517043694E-192	5.71633838391031E-189
S100A8	3.77736264395368E-142	4.3168959415984E-139
TPT1	8.98806068292155E-15	3.90070456346792E-13
TYROBP	1.59502718816892E-304	7.29140095284952E-301
UBA52	1.4473214866036E-08	3.00735861625482E-07
RN7SK	0.083264383617817	0.201996772852421

scRF

perc99 modification *max-change* signature of 36 genes on the CD8 T cell type with *scRF* among which 19 are not significantly different.

	p-value	FDR (Benjamini-Hochberg)
ABI3	0.692891040254578	0.867157120464618
ACTN1	0.000172906935561	0.002747677536833
AIF1	1.19078798355504E-47	2.72174440107898E-44
AP1S2	1.37663550356152E-11	1.25861195305618E-09
ARPC3	0.000984534912288	0.010959343983052
CAPG	4.52963932374849E-15	6.8263157896579E-13
CD3G	0.00108111317746	0.011729735851022
CST3	5.61959448674846E-29	2.65748685487132E-26
CTSS	7.52504740163562E-18	1.49563043573958E-15
CYTH4	0.389535595119556	0.650918868218544
DENND6B	0.063010949517733	0.251347341968058
ENO1	0.136283574183205	0.400762159167985
FTH1	1.50766751959608E-24	4.80840752645131E-22
GIMAP4	0.03094920314195	0.153060718315436
GRN	2.31339104228534E-15	3.5647016577417E-13
HLA-DMA	5.65104352427264E-13	6.24987184611895E-11
KLF2	0.135183072092711	0.400762159167985
LGALS2	8.90147880698769E-41	1.22074880359029E-37
LGALS3	0.01977642589091	0.11433975744854
LINC00938	0.672365983044197	0.858629955439809
PGD	8.99123267040837E-05	0.001564793970076
PSMA7	0.438267736673173	0.700431621108949
PTP4A2	0.286226230481173	0.585207833264458
RAD23A	0.244928674057365	0.53915759807748
RP11-220I1	0.65743593953249	0.850563844196163
SEMA4A	0.000709891131129	0.008539865765178
SERPINA1	1.97721159617934E-16	3.43233921898779E-14
SFR1	0.730681882585537	0.891192755049632
SLC30A7	0.435408371337413	0.697080364758497

SND1	0.375602533110971	0.644383642103995
SPAG7	0.079664091929472	0.277494712459417
SPINT2	2.91855448858505E-12	2.85893258974682E-10
TIMP1	1.85036187944303E-12	1.87969354182827E-10
TTC38	0.032496517459464	0.159391001587657
UBALD2	0.218900262471268	0.500166311151445
WDR83OS	0.748143992149645	0.901529379487447

scAnnotatR

List of 12 possible genes for the *negative-aberrant single-gene* attack on *scAnnotatR* on the platelet cell type among which 9 are not significantly different.

	p-value	FDR (Benjamini-Hochberg)
AIF1	0.00040162192089	0.001965682734861
CST3	5.73419784649663E-14	2.26624752930417E-12
HLA-DPB1	1.15911273704204E-14	5.21182691009656E-13
HLA-DRB1	1.00033671146787E-13	3.84275004511777E-12
LGALS1	1.11078145553336E-08	1.52028511788268E-07
LYZ	2.26767928560713E-07	2.36493944660199E-06
MALAT1	5.08650376494787E-07	4.90205991795468E-06
S100A4	0.001058782727789	0.004644960437907
S100A8	0.358132592143858	0.524221407691415
S100A9	0.966195111868821	0.990165876862129
TYMP	0.000371949462099	0.001824361560523
TYROBP	3.79775741495692E-08	4.63366950077573E-07
